# Deviation of International Practical Temperatures from Thermodynamic Temperatures in the Temperature Range from 273.16 K to 730 K

**DOI:** 10.6028/jres.080A.068

**Published:** 1976-10-01

**Authors:** L. A. Guildner, R. E. Edsinger

**Affiliations:** Institute for Basic Standards, National Bureau of Standards, Washington, D.C. 20234

**Keywords:** Gas thermometry, International Practical Temperature Scale of 1968, temperature scale differences, thermodynamic temperatures

## Abstract

The range over which thermodynamic temperatures have been realized by gas thermometry at the NBS has been extended to 730 K. The results are preserved by measuring the corresponding international practical temperatures. The difference between them is expressed as the following polynomial:

$$T/K-T_{68}/K_{68}=-120,887.784/T_{68}^{2}+1213.53295/T_{68}-4.3159552+6.44075647\times 10^{-3}T_{68}-3.56638846\times 10^{-6}T_{68}^{2}$$

which is valid in the range 273 to 730 K.

The difference found and the estimated uncertainties at the three defining fixed points in the range covered are

**Table:** 

*t*(°C)	*T/*K*–T*_68_/*K*_68_	Uncertainty	
		Random (99% confidence limits)	Systematic

100	−0.0252	±0.0018	±0.00054
231.9681	−0.0439	±0.0022	±0.0015
419.58	−0.0658	±0.0028	±0.0028

## 1. Introduction

The National Bureau of Standards’ gas thermometer has been described in numerous papers [1–10]^1^ that give details of many of the important parts of the equipment and of some of the special measurements required. The last two of these papers also give the results of measurements with the gas thermometer from 0 to 142°C. A comparison of these results with most earlier work elsewhere shows there are systematic differences that we ascribe to our more complete elimination of the effects of sorption. It was stated in the last paper (and has been further confirmed by more recent observations) that our gas thermometer system and the helium filling gas were clean enough that any residual effect of sorption on these measurements would probably be negligibly small. Ultimately, it will be rewarding to remeasure these temperatures, when the benefits of further experiences with the gas thermometer can be realized. At present, it is more productive to extend the measurements to higher temperatures, both for their own intrinsic interest, and as a further step leading to the gold point. This paper gives the results of the initial measurements of the difference between thermodynamic temperatures and international practical temperatures, at temperatures in the range from 141 to 457 °C, and combines them with the earlier data [9, 10], reappraised in the light of further information.

## 2. Equipment

The essential elements of the gas thermometer have been described in earlier papers [9, 10]. Briefly, it is a constant volume type comprising a 432.5 cm^3^ bulb of platinum-rhodium alloy in the form of a right circular cylinder connected by small bore 90 percent platinum 10 percent rhodium tubing to a valve. The gas thermometer can be connected through the valve with a diaphragm pressure transducer, so that thereafter equality of the gas thermometer pressure with the pressure in the manometer can be established. The gas thermometer has a counter-pressure arrangement, a narrow annulus about 0.25 mm wide formed around the bulb by a heavy Inconel^2^ 600 case surrounding the bulb.

**Figure 1:**
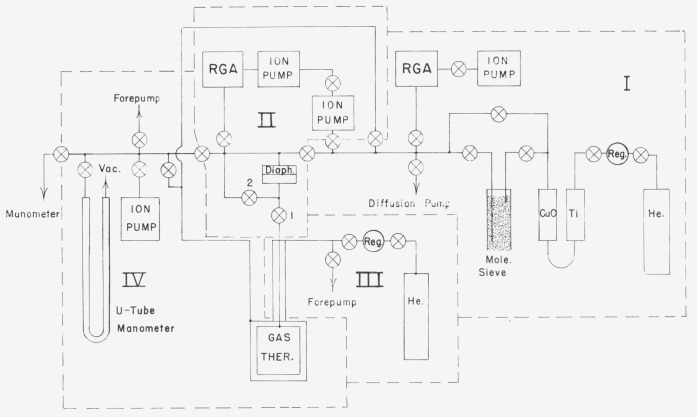
Schematic of vacuum system. Boundaries of Parts I, II, III, and IV, which can be isolated for pumping, are shown by broken lines.

The gas thermometer has been improved by installing more thermocouples to measure the temperature distribution along the small bore tube, and by modifying the gas handling and vacuum equipment.^3^ The new arrangement of the vacuum system, shown in figure 1, allowed the system to be subdivided into four parts (labeled I, II, III, and IV) plus the manometer, so that all the critical sections could be pumped by ion pumps. The subsystems were arranged in such a way as to be able to attain the best vacuum in part II, which included the gas thermometer bulb in direct communication with the residual gas analyzer (RGA).

Two discrepancies from the earlier papers [9, 10] should be noted. The composition of the bulb is not all 80 percent Pt-20 percent Rh as stated in [9], nor all 88 percent Pt-12 percent Rh as stated in [10]; it is some of each. The sheet supplied for the top was found to have been 80 percent Pt-20 percent Rh, but the sheet for the sides and bottom was 88 percent Pt-12 percent Rh. However, the bulb is the same Bulb III referred to previously; the effect of this difference will be considered in the Discussion. Also, the volume of 454.8 cm^3^ given for Bulb III has been re-evaluated as 432.5 cm^3^. The effect of this difference on earlier results will be tabulated in table 4, section 8.

The liquids in the stirred baths were changed from organic materials. The liquid for the bath at 0 °C was changed to water, to which potassium chromate was added as a rust inhibitor, and the liquid for the bath at temperatures from 142 to 457 °C was changed to a eutectic mixture of molten potassium nitrate, sodium nitrate and lithium nitrate. Despite the lesser viscosity of the new fluids, no important change in the performance of the thermostat was observed.

New equipment was installed for the electrical measurements. All resistances of the platinum resistance thermometers (*PRT’s*) were measured with improved speed and precision on an ac bridge [11]. All thermocouple emf’s were measured by a digital voltmeter.

For thermal expansion measurements, a new interferometer furnace, designed for use up to the gold point, was assembled, and it was operated from −25 to 550 °C so that the thermal expansion of samples of the bulb material could be measured. Details of this equipment and the results will be given in a separate paper.

**Figure 2:**
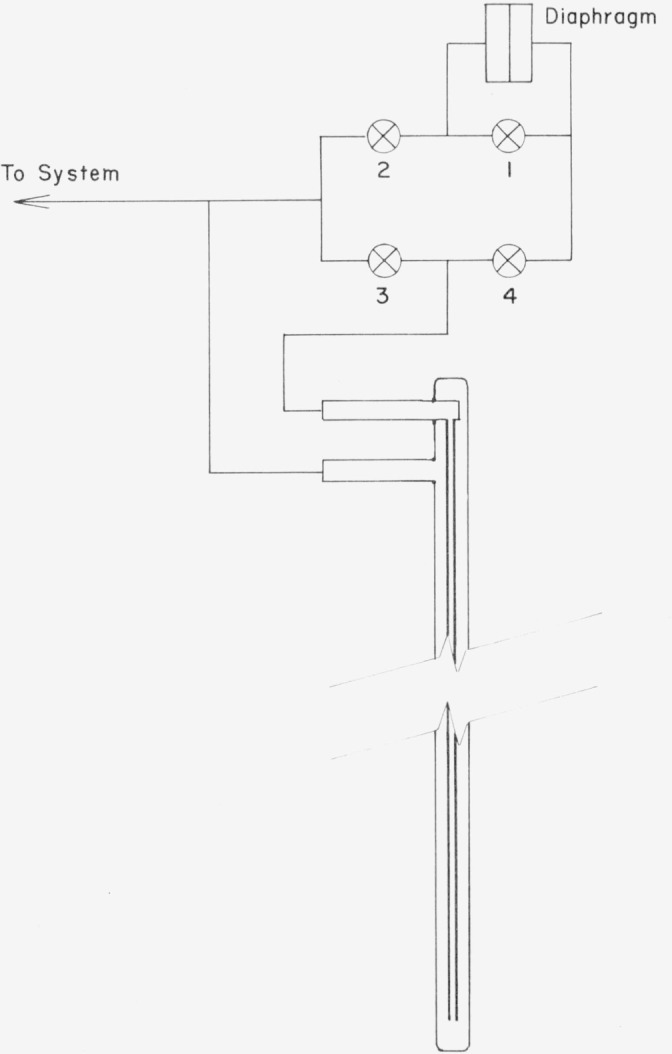
Apparatus for measuring thermomolecular pressure.

The apparatus shown in figure 2 was constructed for the determination of thermomolecular pressure effects. It consisted of two tubes, an 0.8 mm i.d., 1.6 mm o.d. stainless steel tube running inside a 9.6 mm i.d. Inconel tube, connected across a diaphragm pressure transducer. The outer tube was chosen of such a size that it could be inserted into one of the PRT wells of the gas thermometer bulb case to thermostat it in the stirred molten-salt bath. The apparatus was attached to the gas handling system, so that it could be evacuated, and thereafter gas could be introduced. The overall pressure was read with sufficient accuracy on a simple U-tube mercury manometer. The valves in the system were arranged so that when valves 2 and 4 were closed and 1 was open, the null of the diaphragm could be read, and when 1 and 3 were closed and 2 and 4 were opened, the thermomolecular pressure difference could be read on the diaphragm gage.

## 3. Preliminary Considerations

A study of the early results in the extended temperature range showed the need for modifying the experimental procedures. This section presents a discussion of some of those problems and their solutions.

During vacuum bakeout we observed the ubiquity and persistence of hydrogen that is a common experience, and for which there has been a common explanation: that the hydrogen was absorbed in the metal in great quantity and diffused out of it slowly. Perhaps it was a happy accident that we introduced some wet helium into the counter-pressure system at 700 °C, and to our astonishment observed a drastic rise of the hydrogen peak on the RGA scan of the gas thermometer contents within 20 s. Inasmuch as the helium peak did not change, the hydrogen must have diffused through the wall, made of 88 percent Pt-12 percent Rh metal. The diffusion of hydrogen through platinum is covered in the literature, which is ample, and is adequately summarized in Dushman [12]. There is considerable variation in the results, but a typical set of values at 750 °C is as follows:

Hydrogen diffuses through pure Pt at the rate of 0.13 Pa-liter per cm^2^ per min per mm thickness of the metal, with a hydrogen pressure of 10^5^ Pa on one side and a vacuum on the other (the “permeation rate”). Platinum immersed in hydrogen at a pressure of 10^5^ Pa absorbs 0.34 Pa-liters of the gas per cm^2^ per mm thickness.

Both the permeation rate and the solubility for hydrogen vary as *P*^½^, so their relative relationship remains the same at lower concentrations. The inescapable conclusion is that hydrogen initially dissolved in the platinum will quickly be exhausted, and conversely that persistent concentrations of hydrogen observed during vacuum bakeout must be maintained by diffusion through the platinum. The same range of values of diffusion and permeation can be expected for platinum-rhodium alloys. The concentration of free hydrogen in the atmosphere is very small; the only possible sources are water or other hydrogen bearing materials which decompose to release hydrogen. Therefore, we surmised that the problem might be resolved by control of the ambient atmosphere. The furnace was flushed with argon during the bakeout, and a slow flow maintained thereafter. Previously, the partial pressure of hydrogen in the gas thermometer bulb had never been lower than 70 *μ*Pa, but it declined to 20 *μ*Pa at 700 °C when the protective atmosphere was provided.

For the present temperature range up to 457 °C, we found it necessary to change our procedures in three other ways which will be described in the following paragraphs:

### Modification of procedure to avoid creep of the gas thermometer bulb

Above 327 °C, the gas thermometer bulb is subject to creep (non-elastic deformation under small stress). It was discovered from the early results of measurements carried out in the same manner as for the 0–142 °C range. The manometer equipment by its nature permits the accurate realization of only one pressure a day, with one or more experimentally determined values of temperature of the gas thermometer necessary to balance that pressure. Overnight, between measurements, we have always pumped out those portions of the system immediately adjacent to the gas thermometer bulb (parts II and IV) in order to maintain the purity of the system. (The pumpout of section II is also desirable for one of the “integrity checks” described below.) The typical pressure in the gas thermometer bulb at 457 °C was 10^5^ Pa; it was expected that it would deform elastically when the counter pressure annulus (in part IV) was evacuated. However, it was found that gas thermometer temperatures determined by measurements made in descending order agreed with those determined by measurements made in ascending order only up to 327 °C. Above 327 °C, the gas thermometer temperatures differed more as the temperature (and, to some extent, time) increased. We also observed some hysteresis of the gas thermometer bulb after the counter-pressure was restored. It was necessary, therefore, in all subsequent measurements to keep the pressure inside and outside the bulb nearly equal until the bulb temperature was below 300 °C. Because it was more convenient to equalize the pressure by reducing it in the counter-pressure system, all values reported in the paper were derived from runs starting at the highest temperature and proceeding by consecutive steps of about 33 °C to the lower limit.

### Modification of Temperature Measurement of the Dead Space

The effect of the volume in the connecting tube of the gas thermometer, the “dead space,” expressed in kelvins, is about six times as large at the zinc point as at the steam point. The uncertainties of the dead space effect can best be evaluated by examining the combined equation for the dead-space correction, *δt_ds_*. This is

$$\delta t_{ds}=-\frac{T_{1}T_{2}}{V_{0}}\sum_{k}\left(\frac{V_{k_{1}}}{T_{k_{1}}}-\frac{P_{2}}{P_{1}}\frac{V_{k_{2}}}{T_{k_{2}}}\right)$$ (1)

where the summation is over the length of the connecting tube in mm increments, *T*_1_ is the fiducial or reference temperature, *T*_2_ is the measuring temperature, *P*_1_ and *P*_2_ are the corresponding pressures, *V*_0_ is the volume of the bulb at *T*_1_ *V_ki_* and *T_ki_* are the corresponding values of volume and temperature at the *k*th position for state *i*. The element of volume varies between the two states only because of thermal expansion, i.e., *V_ki_=V_k_*(1 *+ β_ki_δt_ki_*), where *β_ki_* is the coefficient of thermal expansion depending upon the position and the state, and *δt_ki_* is the difference in temperature of the *k*th position in the *i*th state from the calibrating temperature of 23 °C. The determination of the volume is thought to be sufficiently accurate [7]; the measurements of the *T_ki’s_* on the other hand required improvement.

We note from the equation that for any region of the dead-space actually at the gas thermometer temperature in both states, the net contribution is zero. The accuracy is increased, then, by maintaining the level of the liquid in the bath as high as possible. Unfortunately, the molten salt, because of low viscosity and low surface tension, could not be confined as readily as the earlier bath fluid. Even at the highest temperature the bath had to be less completely filled or the salt would overflow, and it contracted so much over the range of temperature that the level was appreciably reduced at the lowest temperatures.

Previously the temperatures for the dead space calculations for the steam point were derived on the following assumptions:

- That the lowest junction is at the temperature of the bath.
- That the emf’s of the Pt-10 percent Rh/Pt thermocouples installed for dead space measurement follow the same temperature dependence as was determined for annealed thermocouples of the same spools of wire.
- That the portion of the dead space above the uppermost junction was at the temperature of that junction.
- That the values of the temperature can be adequately interpolated from least squares fits of the observed temperatures to polynomials chosen according to the complexity of the temperature profile.

The consequence of these assumptions is that one may measure all emf’s as differences from the bottom one and then the temperatures can be derived from the calibrations by calculating the equivalent emf of each couple with an ice junction.

Probably the steam point dead space calculation was adequately accurate because its contribution in kelvins was comparatively small (0.034 K), the height of the bath liquid was satisfactory, the gradients were not severe, and the actual thermocouple emf’s were reasonably close to standard table values. However, in this present temperature range, all these factors are less ideally fulfilled. The value of the dead space correction becomes large, partly because the bath liquid level had to be lower; the gradients, of course, were substantial, and as the temperature became high, the deviation of the actual emf’s versus temperature from the table values became important. It is certain moreover that the bottom thermocouple was not adequately immersed. All these factors required that a better measurement and calculation be employed.

Instead of referring the temperatures to the bottom thermocouple, if the temperature of the reference junctions of the dead space thermocouples were established, the error in the dead space calculation could be reduced. This is true because the temperature is then more accurately known than before at the lower temperatures, where the relative effect of a given volume of the dead space is larger; the same discrepancy in emf produces a smaller error in kelvins if it affects the higher temperatures where the thermoelectric power becomes twice as large as at room temperature; and the accuracy of the result then no longer relies upon the bottom couple being at bath temperature. The details of this change will be discussed in section 6.

### Modification in the handling of the platinum resistance thermometers

We found the platinum resistance thermometers to be much more sensitive to shock in the upper range of the temperature investigated. The vibration of the stirred liquid baths caused a significant increase of the resistances of the *PRT’*s above 200 °C (not accountable by the effects reported by Berry [13], so that the procedures were changed to minimize the time during which the thermometers were kept in the bath. The measurements of the *PRT* resistances were made at the end of the time they were in the bath, therefore we used the values of the triple points that were found after each gas thermometer run to calculate their resistance ratios. With such precautions, the triple point values did not increase greatly, being subject to a change of <40 *μ*Ω from work hardening during a measurement period, and being partially restored to their original values by annealing at 425 and 457 °C whenever those temperatures were measured.

## 4. Procedure

The gas thermometer was prepared for operation by vacuum bakeout at 750 °C. After an initial pumpdown to a system pressure of 0.01 Pa by the oil diffusion pump, it was connected with the RGA and isolated from all other parts of the system. It was then pumped only by the ion pump of the RGA, a procedure that gives both the highest concentration in the RGA for the detection of sorbable species, and very clean pumping for the gas thermometer. The entire bakeout furnace was isolated and a protective atmosphere of argon was introduced into it in order to exclude ambient air and prevent the diffusion of water vapor to the hot zone. The pumping was continued until the partial pressure of all contaminating gases was less than 0.1 mPa in the gas thermometer.

The measurements for this paper were made over a period of 15 months during which time the gas thermometer was loaded 10 times and measured at 123 different temperatures. Of the first 51 temperatures measured only 9 are free of creep effects, and of the remainder, the last 43 have the best measurements of the temperatures for the dead space.

The gas thermometer was loaded while being thermostated at the highest temperatures to be measured. The initial measurements were commenced on the following day. At the beginning of a measurement, parts I, II, and III of the vacuum system were isolated but each was being evacuated, part I by its diffusion pump, part II by both ion pumps and part III by its ion pump. The procedure for the measurement of a gas thermometer temperature was as follows:

1. Before loading, or at other times before introducing helium as a pressure transmitting gas, the diaphragm section was isolated by valves 1 and 2, and the rate of pressure buildup was determined over a period of a half hour or more.^4^ The rate varied between the limits of 2.5 to 5 mPa/min. With a total volume of about 100 mm^3^, the relative amount of gas accumulating in the diaphragm compared with the amount of gas in the gas thermometer (for a pressure of 10^5^ Pa) varied between 6 × 10^−12^ min^−1^ and 1.2 × 10^−11^ min^−1^. The measurement is an “integrity check” to ascertain that the diaphragm is neither contaminated nor has a leak from the outside, and further that there is no significant leakage across the seat of valve 1. The same rate was observed whether the gas thermometer was evacuated or filled, hence it indicated no significant leakage occurred across the valve seat, and probably represented mostly, or totally, degassing.
2. At the same time, the heater for the Ti-CuO purification trap was turned on. Within 1 hr, the trap reached its operating temperature (700 °C), as confirmed by measurement with a Pt-10 percent Rh/Pt thermocouple referred to ice.
3. Two integrity checks of the ac bridge and triple point cell were made. First, the ratio of the standard 100 Ω resistor to a 100 Ω *PRT* was read to assure proper thermostating of the standard. Then the ratio of the resistance of a standard *PRT* immersed in a triple point cell to the standard resistance was determined on the ac bridge. This thermometer was treated with the utmost care, so that the ratio was reproducible within less than 1 part in 10^7^ over a period of a month, a fact that tended to confirm that the system was functioning properly. Over longer periods, the observed small changes in the ratio could have occurred because of drift in the resistances of either the standard resistance or the thermometer.
4. The ratios of the resistances of our 3 measuring *PRT’*s to the standard resistor were determined at the temperature of the triple point of water.
5. The MacLeod gage, monitoring the vacuum in the upper cell of the manometer was read (typically 0.15 mPa or less) (an integrity check).
6. The ion pump currents were recorded (an integrity check). The RGA ion pump, open to part II, could be expected to have a current of 0.5 *μ*a, equivalent to 0.2 *μ*Pa pressure.
7. The Dewar around the molecular sieve (#13) trap was filled with liquid nitrogen, and when the temperature of the Ti-CuO trap had reached 700 °C, part I of the vacuum system was isolated and helium was slowly passed through the traps into the lines.
8. After isolating the ion pumps, helium was admitted to sections 2 and 4, until the pressure as indicated on the U-tube manometer was higher than the pressure in the manometer lines. Invariably, the gas flow was controlled to proceed in the direction from I to II and IV so that no backflow of helium would occur from other parts of the gas thermometer. Finally the manometer gas line was opened and the system pressure adjusted. When it was nearly at the pressure needed, the motor of the valve allowing communication of mercury between the upper cell and the lower cells of the manometer was turned on. The length of time it was necessary to open the valve before flow commenced was recorded (an integrity check). Final adjustments of the amount of gas in the system were made, the mercury valve was opened fully and the level of mercury in the cells was checked. The mercury level was remarkably stable over periods of weeks (within about 2 *μ*m), a fact that depends not only upon a stable temperature but also upon a stable contact angle with the wall. After any needed adjustment of mercury level, the gas pressure was controlled by “thermal injection”, governed by feedback from the capacitance bridge to maintain the pressure at the manometer setting.
9. After reading the diaphragm zero, we closed the by-pass valve and opened the valve that connected the gas thermometer valve with the diaphragm. The pressure in the gas thermometer was made equal to the pressure in the manometer by adjusting the temperature of the liquid thermostat. When the pressures were about equal, the gas thermometer valve was closed, the by-pass valve opened, and the null of the diaphragm reread. The valves were then reset to reconnect the diaphragm to the gas thermometer so that any small difference between the pressure in the gas thermometer and the pressure in the manometer system could be observed and recorded. Then the gas thermometer was again isolated and the null reread.
10. The resistances of the *PRT’s* in the gas thermometer bulb case were read, as well as that of the manometer reference *PRT*, then the 5 thermocouples of the manometer and the 16 thermocouples of the dead space.
11. Another set of null-pressure-pressure-null measurements was made with the diaphragm.
12. Data also included were the time, the values of the gage blocks used in the manometer, and the regulator bridge and control settings.
13. The resistances of the platinum resistance thermometers were measured at the temperature of the triple point of water.

## 5. Equations and Calculations

The calculations for the gas thermometer temperatures can be made “symmetric”, in that the equations can be devised so that the same calculation is performed for each measurement including that of the reference state. The details are given in [9]; the essentials are repeated here. We calculate a quantity designated *Z* that is an “augmented pressure”, expressed in cm of Hg at 20 °C, where the thermal expansion, the pressure head and the dead space effects are accounted for. It is

$$Z_{i}=h_{0i}(1+W_{i}),$$ (2)

where *i* refers to the *i*th measurement, *h*_0_*_i_* is the height of the gage blocks used in the manometer, and

$$W_{i}=\pi_{i}+\beta t_{i}+\pi_{i}\beta t_{i}+[(T_{i}+B_{i}P_{i}/R)/V_{0}][\sum_{k}V_{k}(1+\beta_{ki}\delta t_{ki})/T_{ki})].$$ (3)

In the order of the terms in eq (3), the significance of the symbols is as follows:

$$\begin{array}{l} \pi_{i}\;\text{is the pressure head and is given by}\\ \pi_{i}=\sum_{k}Mgl_{k}/(RT_{ki}) \end{array}$$ (3a)

where *M* is the molecular weight of the gas, *g* is the acceleration due to gravity, *R* is the molar gas constant = 8.3137×10^6^ cm^3^ Pa mol^−1^ *K*^−1^ *T_ki_* is the thermodynamic temperature for the *k*th element of length and *i*th measurement, and *l_k_* is the increment of length.

The volume thermal expansion coefficient of the bulb, *β*, is derived from

$$1+\beta t_{i}={\left(1+{\bar{\alpha }}_{0}{\;}^{t}\right)}^{3},\;\text{and}\;{\bar{\alpha }}_{0}{\;}^{t}\frac{1}{l_{o}}\frac{\Delta l}{t}$$

as determined from samples of material of the bulb of length *l*_0_ at 0 °C. The term *π_l_β_i_* is negligible.

The remainder of eq (3) is the deadspace term. *T_i_* is the thermodynamic temperature of the bath, *B_i_* is the second virial coefficient of the thermometric gas at *T_i_ P_i_* is the pressure of the *i*th measurement, *R* is the molar gas constant, *V_o_* is the volume of the gas thermometer bulb at 0 °C, *V_k_* is the volume of the *k*th element of the connecting tube at 23 °C, *β_ki_* is the thermal expansion coefficient of the volume of the tube at temperature *t_ki_*, in the *k*th position during the *i*th measurement, and *δt_ki_=t_ki_*−23 °C. This same temperature *t_ki_*, is expressed as an absolute thermodynamic temperature for *T_ki_* in the denominator. The quantity *Z_i_* is also given by the expression

$$Z_{i}=Z_{i0}+\delta Z_{i}$$ (4)

where

$$Z_{i0}=\frac{n_{0}R(T_{i}+B_{i}P_{i}/R)}{\rho_{0}gV_{0}},$$ (4a)

with *n*_0_ the number of moles of gas, *ρ*_0_ the density of mercury at 20 °C, and other quantities as defined before. *δZ_i_* is a term for thermomolecular pressure, and any variation of the pressure head in the manometer line from the level of the gas thermometer in the colder thermostat. We have calculated an “approximate gas thermometer temperature”, *T*′*_GT_*, from the equation

$${T^{\prime }}_{\text{GT}}/{K^{\prime }}_{\text{GT}}=T_{i}/K\left(Z_{j}Z_{i}\right).$$ (5)

^5^

Then the gas thermometer temperature, *T_GT_* was derived by correcting *T′_GT_* for the effects of thermomolecular pressures and differing pressure heads, so that

$$T_{\text{GT}}/K_{\text{GT}}={T^{\prime }}_{\text{GT}}/{K^{\prime }}_{\text{GT}}+\delta t_{p}/K=T_{i}/K(Z_{j0}/Z_{i0}).$$ (5a)

We can substitute the expression for *Z_j_*_0_ and *Z_i_*_0_ from 4a into 5a, and then because *ρ*_0_, *g*, and *V*_0_ are constants, and *n*_0_ is maintained constant, we get

$$T_{\text{GT}}/K_{\text{GT}}=T_{j}/K+([B_{j}-B_{i}(P_{i}/P_{j})(T_{j}/T_{i})]P_{j}/R)/K$$ (6)

with additional, insignificant second and higher order terms. Thus the gas thermometer temperature is equal to the thermodynamic temperature plus a term for the effects of gas imperfection. Substantially

$$(P_{i}/P_{j})(T_{j}/T_{i})=(1+kt_{j})=\text{Const}.,\;\text{for}\;t_{j}\;\text{constant},$$ (7)

so that *T_GT_/K_GT_*=(*T_j_+aP_j_*)/*K*. Thus by determining *T_GT_/K_GT_* at a constant pressure ratio but differing pressure ranges, *T_j_/K* may be found as the intercept of the straight line of *T_GT_/K_GT_* versus *P_j_* and the difference of the second virial coefficients can be found from the slope.

**Figure 3:**
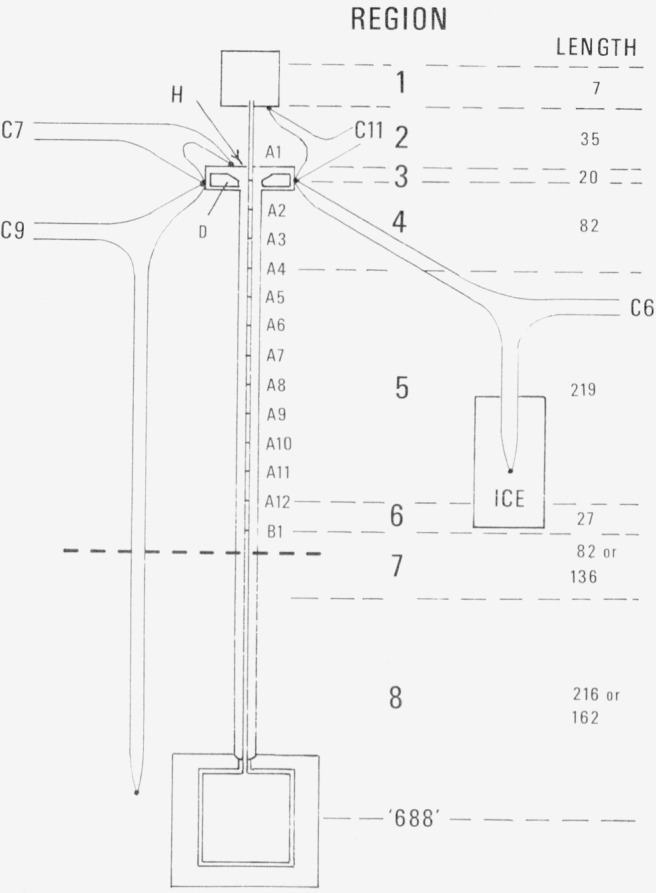
Thermocouple installation for measurement of the temperatures of the pressure head and dead space. The unit of length=0.929 mm.

The calculation is made by computer in a series of programs:

1. The international temperatures of the gas thermometer and the manometer reference station are calculated by a program in Fortran IV named BRIDGE. This program is given in appendix I, with a file of the calibration constants *A* and *B* of the *PRT’s* (TCAL75). A calibration on the ac bridge at the zinc, tin and triple points was made in June 1974, and the last gas thermometer measurements included in this paper were made in September of the same year. (The exact time does not appear to be important. Starting before the measurements were commenced, and extending to a time one year afterward, 4 sets of the thermometer calibrations have been shown to give a reproducibility of values at the temperatures of the fixed points within a standard deviation of 0.45 mK.)
2. The effect of the pressure head is calculated by a program in Basic named PYE. This program is given in appendix 2, and the subprogram LSFIT1 is given in appendix 3. In this program, the the treatment of the temperature distribution is made as described in section 6. The international temperatures, *t*_68_, were determined from the emf’s of the thermocouples for the locations shown in figure 3. A correction was applied to *t*_68_ to give the thermodynamic temperature, *t*_th_, and this in turn was converted to a value of reciprocal absolute temperature, *τ*. The volume of the gas in the constant volume valve and the first 6 mm of tubing within the valve were at the temperature of the valve. It is expected that the good thermal contact provided by the vacuum seal between the tube and the top of the header, H, brought the temperature of the tube substantially into agreement with the temperature of the header at that point. Between the valve and the top of the header (33 mm), the values of r were interpolated linearly, and similarly between the top of the header and the position of the thermocouple *A*1 (19 mm). The length of the tube above the top of the header varied by an amount *D2*, which reflected the differential expansion between the connecting tube of the gas thermometer and its Inconel suspension. (Direct measurements confirmed the calculated value of *D2* within experimental error.) The thermocouples *A*1 …, *A*12, and *B*1 on the other hand terminated close to the connecting tube but were independently suspended, being installed in a harness that was self-supporting and through which the tube could move. The expansion of the thermocouple harness was included in the thermal expansion term for the tube.
  In order to interpolate the reciprocal temperature between the thermocouple locations, a quadratic equation, *τ=B*(1)+*B*(2) *I+B*(3) *I*^2^, was fitted by least squares to the first four pairs of values of *τ* versus position, *I*, for *A*1 *… A*4 (76 mm). A polynomial giving *τ* as
  $$\tau =B(1)/I^{2}+B(2)/I+B(3)+B(4)\;I+B(5)I^{2}$$
  was fitted by least squares to the 9 pairs of reciprocal values of temperature versus position for *A*4…*A*12 (203 mm). In the interval from *A*12 to *B*1 (25 mm) *τ* was linearly interpolated. If the difference in emf between *B*1 and the calculated value for the temperature of the bath (the level of which was near or not more than 20 mm below *B*1) was sufficiently large (>0.1 mV), the difference of the corresponding reciprocal absolute temperatures was linearly interpolated over the next 136 units of length (126 mm), and if <0.1 mV, over 82 units of length (76 mm). The balance of the length of the connecting tube, and an additional length up to the center of the gas thermometer bulb, for a total length count of 688 (639 mm) was included in the calculation. The unit of length was 0.929 mm, which arose as a chart coordinate in measuring the tube diameter. The quantity *π_i_* is calculated as:
  $$\pi_{i}=\frac{Mg}{RK}\sum \frac{l_{k}}{T_{ki}}(1+\alpha_{ki}\Delta t_{ki}),$$
  as given in eq (3a), with the added definition that *α_ki_* is the linear expansion coefficient of the *k*th element for the *i*th. measurement, and Δ*t_ki_=t_ki_−*23. The output of the program gives the notebook reference number (*Sϕ* is the notebook and page, *S*1 the assigned run number), the value of *π_i_* in ppm, and the displacement *D*2 in mm.
3. The calculation of the deadspace effect depends upon the evaluation of Σ*_k_V_ki_/T_ki_* and involves exactly the same temperature calculations. They are associated with values of the volumes over the lengths from *k*-1 to *k*. The calculation is performed by computer with a program in basic named DEDSPS, given in appendix 4. A file of chart readings for the diameter of the tube is a part of this program, that also required the entry of *D*2 from the PYE calculation. The thermal expansion of the tube and of the thermocouple locations is combined in the term (l+*β_ki_*Δ*t_ki_*). The output of the program gives the notebook and run reference numbers and the value of Σ*_k_V_ki_/T_ki_*, designated V*TAU.
4. The value of *Z* is calculated by a program in Fortran IV named SUMMA, which is given in appendix 5. The calculation of the pressure is derived from eq (27) of [5], and is expressed in cm of mercury corrected to 20 °C. The output gives the notebook and run reference numbers and the value of *Z* in cm.

## 6. Determination of Parameters

Numerous calibrations and special measurements are involved in the gas thermometer calculations. Some of them have been mentioned in earlier sections, but they will be summarized here.

### 6.1. The Gas Thermometer Pressure

The value of pressure used in the gas thermometer equation is the sum of the pressure calculated by eq (27) of [5] plus the difference measured at the diaphragm. The height of the mercury column was determined by the length of the calibrated gage blocks. The density of the mercury was adjusted for its variation in temperature from 20 °C. We used the values of the thermal expansion of mercury published by Beattie et al. [14]. There is an offsetting effect of expansion of the gage blocks; the value in SUMMA reflects the difference between the volume expansion of mercury and the linear expansion of chromium carbide. The small values of the copper-constantan difference thermocouple readings for the manometer cells and for the main mercury column were converted to temperature differences by use of a “standard value” of the thermoelectric power, 40.5 *μ*V/K [15], and the reference temperature was measured by a calibrated standard platinum resistance thermometer.

In order to calculate the pressure differences from the gage readings of the diaphragm, its sensitivity was determined from the pressure changes calculated to have resulted from imposed changes of gas thermometer temperature.

### 6.2. The Thermal Expansion of the Bulb

The term in the gas thermometer equation second in importance to the pressure ratio is the term for the thermal expansion of the bulb. The thermal expansion coefficients of both 80 percent Pt-20 percent Rh and 88 percent Pt-12 percent Rh have been carefully measured from −25 to 550 °C. The constants entered into SUMMA are those for the thermal expansion of 88 percent Pt-12 percent Rh where the length ratio is expressed as a polynomial,

$$L/L_{0}=1+C_{1}t+C_{2}t^{2}+C_{3}t^{3}+C_{4}t^{4}+C_{5}t^{5}.$$ (8)

The estimate of the standard deviation of a predicted point varies from 0.08 ppm in mid range to 0.14 ppm at either extreme. The estimate of the residual standard deviation of 0.14 ppm is consistent with our expectation of the average imprecision of a single measurement.

### 6.3. The Deadspace

The values used in the deadspace calculations were obtained from various sources. The volume of the bulb was calculated from its dimensions except for the last gas load. In the bakeout preceding the final set of measurements, the bulb was deformed by an external overpressure. When the bulb was removed from the apparatus, its changed volume was determined from the contained weight of water to be 4.2103×10^5^ mm^3^ at 0 °C, and this value was used in the calculations for the final set.

To determine the volume of the tube, its diameter is required as a function of position. This was determined as described in [7] with an estimated total uncertainty that is insignificant in the final results.

Temperatures were derived from thermocouple readings for the calculation of *π* and *Vτ*. It may be useful to remark in advance that considerable error in the thermocouple values will not significantly affect the accuracy of the final results. The final arrangement illustrated in figure 3 was applicable to the last 30 states, where thermocouples were added about the header, H. The couples *C*6, *C*7, and *C*11 comprise copper-constantan legs (Type T), and *C*9 has Pt-10 percent Rh/Pt legs (Type S). The couple *C*6, with its reference junction at 0 °C, was used to measure the temperature of the side of the header, and inferentially of the reference junctions of *A*1 … *A*12, and *B*1, because it is expected that the temperature of the reference ring D is nearly the same as the side of the header. The difference couples, *C*7 and *C*11 were used to determine the increment of temperature between the top and side of the header and between the end of the tube at the constant volume valve and the side of the header, respectively. *C*9 was a difference couple running between the bath and the side of the header. It was not needed for the last 30 states, but was a necessary link with earlier measurements to evaluate the earlier data. All copper-constantan readings were converted to temperatures by use of a standard table [15]. The temperatures determined by all Pt-10 percent Rh/Pt thermocouples were evaluated from calibrations of similar couples which could be represented by a quartic,

$$E=B_{1}t+B_{2}t+B_{3}t^{3}+B_{4}t^{4},$$ (9)

with a standard deviation of the values at each interval of 25 °C up to 500 °C of 0.47 *μ*V when *B*_1_ = 5.45846×10^−3^ *μ*V/°C, *B*_2_ = 1.13497×10^−6^ *μ*V/(°C)^2^, *B*_3_ = −1.52447×10^−8^ *μ*V/(°C)^3^ and *B*_4_=9.06033×10^−12^ *μ*V/(^0^C)^4^. When the emf of the Pt-10 percent Rh/Pt thermocouple *B*1 was measured at 400 °C (under special conditions where the depth of immersion was thought to be adequate), however, it was found to differ from the equation by −28 *μ*V; the cause of the difference appeared to be work-hardening of the platinum leg during installation that was not subsequently relieved by annealing. This difference was assumed to be typical of all the deadspace couples and was taken into account in the gas thermometer calculations by multiplying all values of *A*1 … *A*12, and *B*1 by 1.00895.

In an earlier arrangment of the thermocouples applicable to the preceding 42 states, the *C*9 junction was located on the top of the header, and there was no *C*6, *C*7, nor *C*11 couple. Furthermore, *C*9 was calibrated at 400 °C and found to differ from the value calculated from eq (9) by −51 *μ*V. Consequently, the measured emf’s for *C*9 were multiplied by a factor, 1.01644, to account for the difference.

**Figure 4:**
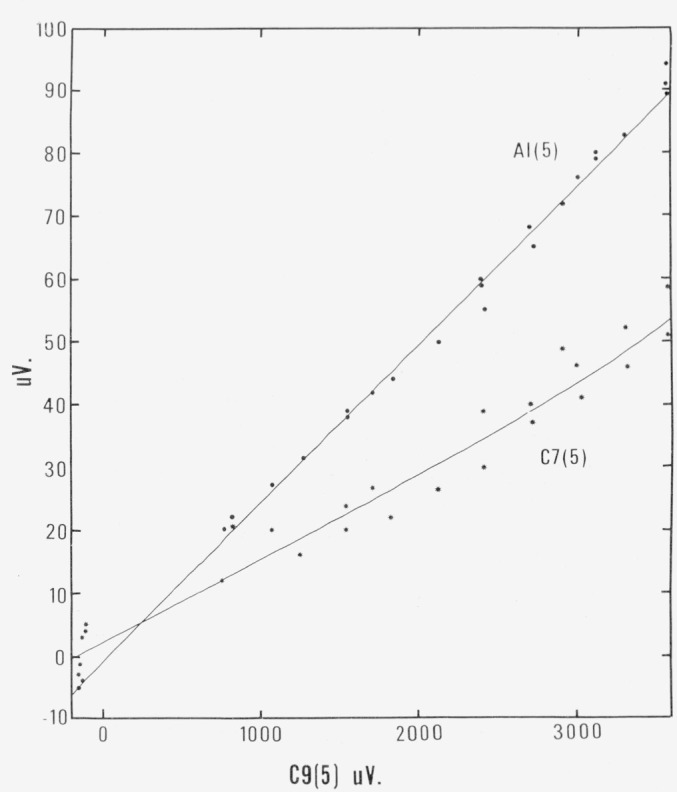
Values of *A1*(5) and *C7*(5) as a function of bath temperature, given by *C9*(5).

To carry out the calculation described in the preceding paragraphs it was necessary to predict values of *A*1(5), *C*7(5), and *C*11(5), for the earlier measurements. This could be done from the information available from later measurements. In one switch position, the thermocouple emf’s could be read with respect to *B*1 as the reference junction (position 4) and were designated as *A*1(4), etc. In another switch position, the thermocouples were referenced to their own junctions with copper (position 5), and measurements with this reference were designated as *A*1(5), etc. Because the temperature of the reference ring is uniform, *A*1(5) – *A*1(4) = *B*1(5). Position 5 of the switch was used to read the thermocouples for the last 30 states. As shown in figure 4, the values of *A*1(5) and *C*7(5) are well-behaved functions of the bath temperatures as expressed by *C*9(5). As for *C*11(5), its values depend not only upon the temperature of the bath, but also upon the temperature of the room and of the plate directly below it which is cooled by tap water. For constant room and cooling water temperatures, *C*11 decreases linearly as the bath increases in temperature, over a range of about 40 *μ*V. The cooling water temperature varies seasonally from less than 8 °C in the winter to over 20 °C in the summer; this explains the large differences in *C*6(5). The 40 *μ*V range of *C*11(5) tends to be more negative as the cooling water temperature increases. The values of *C*11 (5) used in table 2 were derived on the basis of a linear variation from 40 or 41 *μ*V at 0 °C to 0 *μ*V at 457 °C. A uniform change of 20 *μ*V in the values of *C*11(5) causes an error in the calculated gas thermometer temperature varying from 0.2 ppm at 140 °C, up to 0.5 ppm at 457 °C. Therefore high accuracy in predicting these *C*11(5) values is not a requirement.

To summarize: For all the earlier data for which only values of *C*9(5) referenced to the top of the header are available, we find the temperature distribution along the tube by the following steps:

1. Read *C*7(5) and *A*1(5) from figure 4. Calculate 6711(5) by *E*−40 (457−*t_bath_*)/457 *μ*V.
2. Find the emf of a standard couple referred to ice for the top of the header in *μ*V for Pt−10 percent Rh/Pt from *V_ϕ_* (*t_bath_*)−*E_ϕ_×*1.01644, where *V_ϕ_* is calculated from the quartic equation and *E_ϕ_* is *C*9(5).
3. Deduct *C*7(5) × 0.125 *μ*V to account for the difference in emf corresponding to the difference in temperature between the top of the header and the aluminum reference ring. The equivalent emf for *C*6(5) can be found if desired.
4. Add *A*1(5) × 1.00895 to give a synthesized value of emf referenced to ice.
5. Find *t_A_*_1_ from the derived emf. Similarly, the temperatures can be found for all other positions including *B*1.

### 6.4. Determination of Thermomolecular Pressures

Using the apparatus developed for this purpose, and described in section 2, we measured some thermomolecular pressure differences for helium at temperatures of 140, 260, 374, and 457 °C at pressures varying from 2.5×10^3^ to >1×10^5^ Pa, The results were correlated by an equation

$$\bar{p}\Delta p=C_{1}\left[{\left(\frac{T_{2}}{T_{0}}\right)}^{2n+2}-{\left(\frac{T_{1}}{T_{0}}\right)}^{2n+2}\right],$$ (10)

based on Weber’s treatment [16], where the product of the average pressure $\bar{p}=(p_{1}+p_{2})/2$ and the pressure difference Δ*p* is expressed in relation to *T*_2_, the upper temperature; *T*_1_, the lower temperature (296 K); and *T*_0_, the reference temperature (273.15 K). The reference temperature is involved only in that the exponent *n* is defined by the assumption that the temperature dependence of the viscosity of helium can be expressed by

$$\eta /\eta_{0}={\left(T/T_{0}\right)}^{n+1/2}.$$ (11)

For the temperature range 0 °C <*t* <460 °C, *n* was evaluated as 0.145. A least squares solution gave *C*_1_ = 3738 Pa^2^, with a standard deviation of 1.8 Pa^2^. The average deviation of the measured values from the equation is 1.8 percent, and the maximum deviation is 3.4 percent. Because *C*_1_ varies inversely as the square of the diameter of the tube, the measured value of *C*_1_ was modified to account for the smaller diameter of the gas thermometer tube by a factor (0.823/0.904)^2^, or *C*_1_=3098 Pa^2^.

## 7. Tabulation of Results

The measurements which will be presented in subsequent tables are subdivided according to experimental groupings labeled Set A to Set I. More information by group is given in table 1.

The values of the calculated quantities for the states of the gas thermometer are tabulated in tables 2 and 3. The results have been divided into those in table 2, in which temperatures of the header were derived by calculation, and those in table 3 for which the thermocouple emf’s were experimentally observed. The deviations of international temperatures from gas thermometer temperatures were calculated from the equation

$${T^{\prime }}_{GT}/{K^{\prime }}_{GT}-T_{68}/K_{68}=T_{i}/K(Z/Z_{i})-T_{68}/K_{68}$$ (12)

where *T_i_* is the absolute temperature of the chosen reference state with the corresponding value of *Z=Z_i_*. The difference *T′_GT_/K′_GT_ − T*_68_*/K*_68_ was further modified for the effect of the lower level of the gas thermometer bulb in the high temperature bath; the smaller pressure head requires a decrease in all the gas thermometer temperatures of 0.5 ppm except those measured in the low temperature bath. These changes are smaller than the thermomolecular pressure effects that were calculated by eq (10), and that were included in the results in column 15, headed *T_GT_/K_GT_ − T*_68_*/K*_68_.

## 8. Review of Previously Published Results

The results reported in our last paper [10] were calculated by programs similar to those used for the present data. The PYE and DEDSPS programs derived temperatures in the manner described as our “original method,” and the SUMMA program differed in the form of the equation and the constants used for the thermal expansion of the bulb, with a trivial effect on the results. Both of these differences can be expected to produce a difference less than 0.5 mK from the present approach. The original data and some of the quantities derived from intermediate calculations are given in table 4, columns (1) to (10), and the values reported previously are given in column (11). As stated in section 2, the actual volume of the bulb was found to be different from the value used in the original SUMMA program. The change necessitated by correcting the volume of the bulb is given in column (12). A further adjustment for the effects of thermomolecular pressure, as determined by eq (10), is given in column (13).

## 9. The Effect of Gas Imperfection

When the numerical differences, *T_GT_*/*K_GT_*−*T*_68_/*K*_68_, for the same temperature obtained at nearly constant pressure ratio but over different ranges of pressure are extrapolated vs the upper pressure to zero pressure, the intercept is *T/K*−*T*_68_/*K*_68_. Alternatively, if the equation of state for 1 mol of gas is considered in the form *pV=RT+Bp*, the thermodynamic temperature ***T*** is related to a gas thermometer temperature *T_GT_* by

$$T/K=T_{GT}/K_{GT}-\{[B_{j}-B_{i}(p_{i}/p_{j})(T_{j}/T_{i})]p_{j}/R\}/K$$ (13)

where *j*’s refer to the measuring state, and *i*’s to the reference state, and *R* to the molar gas constant. Considered in terms of this equation, the accuracy with which the thermodynamic temperature can be found by extrapolation will be optimized when the range of *p_j_’s* is large, so long as the lowest *p_i_* is still large enough to be realized with the same relative accuracy as large values. However, at low pressures, thermomolecular pressure effects become important. For instance, two pressure ranges were used at 100 °C. For *p_j_* = 1×10^5^ Pa, the combined thermomolecular pressure effects for *p_j_* and *p_i_* are 0.34 ppm, while for *p_j_*= 18374 Pa, the combined thermomolecular pressure effects are 10.6 ppm. For all values in Sets A, B, C, G, H, and I, all *p_j_’s* were above 5×10^4^ Pa, so that the thermomolecular pressure affects the gas thermometer temperature by at most 2 mK.

Unfortunately, the number of measurements at varying pressure ranges was insufficient to permit precise extrapolation to zero pressure. An alternative to extrapolating is to calculate the effects of gas imperfection from independently determined second virial coefficients of helium. Our original decision to rely upon extrapolation involved our appraisal that the use of virials from the literature would not give as accurate thermodynamic temperatures. This remains our conclusion, provided there are sufficient gas thermometer measurements;^6^ in the interim we are compelled to use literature values of virials to derive thermodynamic temperatures, but with more uncertainty than we would expect when our own measurements are sufficiently complete.

Second virial coefficients calculated from compressibility measurements are probably more accurate than from other sources; the Burnett method [17] is probably the most accurate of the experimental compressibility techniques. We have chosen a set of virial coefficients based upon the values obtained at the NBS by the Burnett method for temperatures up to 150 °C [18]. The values at higher temperatures are those of Yntema and Schneider [19], who also used the Burnett method. The interpolation and smoothing were made consistent with acoustic results of Gammon and Douslin [20]. The results from these three sources differ from each other substantially more than their respective estimated uncertainties. The values used and the equation derived by a least squares fit to the data are given in table 5.

$$B/({\text{cm}}^{3}\text{mol})=11.9967-4.48574\times 10^{-3}t+1.46724\times 10^{-6}t^{2}.$$ (14)

The effects of gas imperfection increase with increasing temperature and, of course, with increasing pressure. It amounts to 0.0227 K for *T*_2_=730 K and *p*= 10^5^ Pa, and only 0.0002 K for *T*_2_=293.81 K and *p* =14500 Pa. The multiplying factor on *B*_1_, *f =p_i_/p_j_*·*T_j_/T_i_*, is always greater than unity above 0 °C, therefore the sign of the difference of the virials *B*_2_*−fB*_1_, is always negative. Consequently, the thermodynamic temperature calculated from eq (13) is always greater than the gas thermometer temperature.

The results corrected for gas imperfection are given in the final column of tables 2, 3, and 4, and represent the difference between international and thermodynamic temperatures. An equation giving the deviation as

$$T/K-T_{68}/K_{68}=A_{1}/T_{68}{\;}^{2}+A_{2}/T_{68}+A_{3}+A_{4}T_{68}+A_{5}T_{68}{\;}^{2}$$ (15)

was fitted to all the values in tables 2, 3, and 4 by least squares. The constants found are given in table 6.

**Figure 5:**
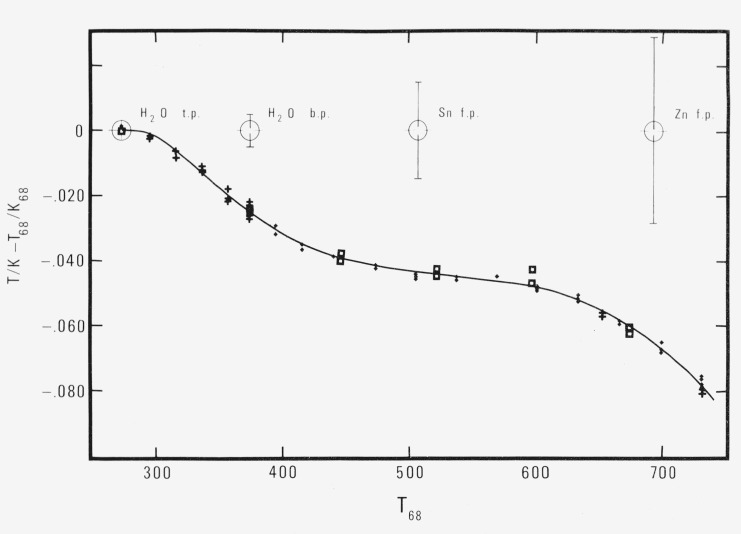
Deviation of International Practical Temperatures from thermodynamic temperatures in the range from 273 to 730 K. The estimated uncertainties at the defining fixed points published in the original text of the IPTS-68 are shown by the error bars. Key: □ high pressure range, ♦ medium pressure range, + low pressure range.

The estimate of the standard deviation of the fit,

$$S=\sqrt{\frac{\sum \delta x_{1}{\;}^{2}}{N-M-1}}$$ (16)

where *N* is the number of points and *M* is the number of constants, is 1.52 mK. The estimate of the standard deviation of a predicted point varies from 0.75 mK near the triple point, to 0.34 mK in the middle of the range, to 0.46 mK near the upper end. The values and the calculated curve are shown graphically in figure 5. The least squares computer program is given in appendix 6 and the computer printout of the least squares solution is given in appendix 7.

## 10. Study of IPTS Realization Above 300 °C

**Figure 6:**
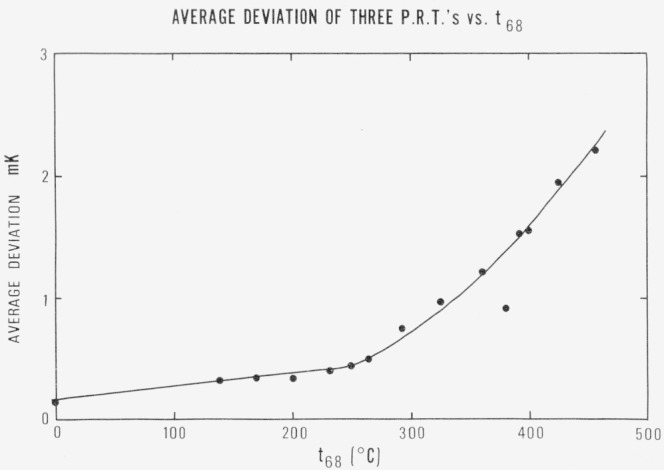
The average deviation of three *PRT’s* versus the average IPTS temperature.

Above 200 °C, the values of temperature determined from 3 standard *PRT’*s differed from one another at the same temperature by more than might be expected from the calibration results. The average deviation from the mean of their temperature values versus *t*_68_ was derived from columns 2, 3, 4, and 5 of tables 2, 3, and 4 and is shown in figure 6.

Subsequent to all the reported gas thermometer measurements, we carried out special measurements at 408 and 414 °C to study this apparent contradiction. It has already been noted that the triple point resistances of the thermometers usually increased with time of immersion in the stirred liquid thermostat of the gas thermometer. But we have also observed that the triple point resistance, if already in substantial excess of its calibration (annealed) value, decreases initially when placed in the thermostat at 457 °C. We now interpret this fact as resulting from a balance between the rate of increase of the resistance from work hardening of the platinum by the mechanical acceleration from the stirring and the rate of decrease of the resistance by annealing of the platinum at 457 °C. At somewhat lower temperatures (350 to 420 °C) the rate of increase from work hardening remains high, but the annealing rate is substantially smaller, so that after the thermometers had been in the thermostat, a rise in the triple point resistance was observed. We found the triple point resistances to increase progressively with succeeding periods in the thermostat at 408 °C, and, although the average temperature remained nearly constant—both in fact and as calculated from the average of the values determined from the thermometers—the average deviation from the mean increased successively from 1.63 mK to 1.89 mK to 2.13 mK. When annealed before each measurement, the average deviation from the mean became 0.65 mK and remained essentially constant with further cycles. The precision of the values of *t*_68_ was the same after ½ h anneals as after 2½ h anneals. Although we also used different annealing temperatures, the best choice, on the basis of Berry’s isochronal step depression results [13], is probably 450 °C. Some conclusions one may draw:

- Platinum resistance thermometers become very sensitive to mechanical shock at temperatures in excess of 350 °C; and up to 457 °C, the calculated temperature determined by them tends to drift in value with progressive work hardening. For relatively minimal lengths of exposure these increases were limited to steps of resistance corresponding to less than 0.30 mK.
- The direction of change of the resistance of a *PRT* at the zinc point from its annealed value because of work hardening is unpredictable on an a priori basis. For the thermometers we happened to use, the average of the values of *t*_68_ calculated from the measurements were a good approximation (within 1 mK) of the true value of *t*_68_, whether determined from the annealed thermometers, or from thermometers of the same group which exhibited the effects of work hardening.
- The values of *t*_68_ calculated for a given temperature as measured with a group of thermometers kept as closely as possible in an annealed condition, are in good agreement with one another. The best choice of annealing temperature is probably 450 °C, and at least for thermometers only slightly work hardened, a half-hour of annealing is sufficient. We believe under our conditions of measurement, the thermometers should be annealed each time before measurement is made, and, as has become our practice, the time during which the thermometers are subjected to mechanical acceleration should be kept as short as possible.

Other causes of measured temperature difference, investigated and found insignificant, were possible temperature gradients in the gas thermometer bulb case and possible imprecision of the thermostat temperature.

## 11. Estimation of Total Uncertainty

The stated total uncertainty consists of the limits, at 99 percent confidence level, for the random errors; and the systematic errors, estimated conservatively enough to warrant about the same confidence level. We discriminate between errors which fall into three categories:

- Sources of random error which contribute to the imprecision observed in the results.
- Sources of error which produce a constant bias in the results, but which are estimable in terms of random errors determined from other experiments.
- Sources of “systematic” error which produce a constant bias in the results, but for which there is inadequate experiment and theory to permit their evaluation by accepted statistical techniques.

The estimates of the values of the random errors (*A* and *B*) are expressed initially as one standard deviation.

The error of the reported quantity, the deviation of international temperatures from thermodynamic temperatures, *T/K*−*T*_68_/*K*_68_, can be estimated by combining in quadrature the errors of realizing the international temperatures with the errors of realizing the thermodynamic temperatures. The errors for each depend upon the range of temperature, and for the thermodynamic temperatures, also upon the range of pressures. There are two values of temperature for which a larger number of values was determined—373 and 730 K. We propose to assess the errors at these two temperatures, being values at or near the extremes of the range, as well as to consider the statistical evaluation of the imprecision derived from fitting the curve in terms of the estimate of the standard deviation of a predicted point.

The errors in the realization of the international temperatures are those from:

1. The calibrations. The systems of measuring instruments, fixed points and thermometers is sufficiently exact that for the four standard *PRT’*s used in the measurements, the constants *A* and *B* in the equation
  $$R=R_{0}(1+At^{\prime }+B{t^{\prime }}^{2}),$$ (17)
  determined in four successive calibrations over a period of two years defined the value of the temperature at the zinc point with an estimated standard deviation of the mean of the thermometers of 0.20 mK.
2. The values of *t*_68_ determined by three standard instruments which had the precision of calibration stated in (1) above.

The estimate of the standard deviation of the mean in realizing the international temperature can be evaluated by combination of the estimates of the standard deviations of the mean of the temperature imprecision of the 3 thermometers because of the imprecision of the constants of calibration, *S*_1_; and 3 factors involved in the measurement of a temperature: the imprecision of the thermostat bath regulation, *S*_2_; the imprecision of the measurement of the resistances, *S*_3_, and the uncertainties because of the effects of work hardening discussed in section 10, *S*_4_. These are given in table 7.

The approximate gas thermometer temperature was calculated from eqs (2) through (5a) and the thermodynamic temperature was then found by accounting for the effects of thermomolecular pressure and gas imperfection. The approximate gas thermometer temperature can be expressed in terms of the ratio in a way useful for error analysis as

$$\begin{array}{c} {T^{\prime }}_{\text{GT}}/{K^{\prime }}_{\text{GT}}=T_{1}/K(Z_{2}/Z_{1})\\ =(T_{1}/K)\left(\frac{P_{2}}{P_{1}}\right)\left(\frac{1+\pi_{2}}{1+\pi_{1}}\right)(1+\beta \Delta t)-\frac{(T_{1}/K)T_{2}}{V_{0}}\sum_{K}\left(\frac{V_{K_{1}}}{T_{K_{1}}}-\frac{P_{2}}{P_{1}}\frac{V_{K_{2}}}{T_{K_{2}}}\right). \end{array}$$ (18)

The value of the second term is small relative to *T_GT_*, so that the relative errors of the products in the first term are directly reflected as relative errors in the gas thermometer temperature. In the order of the quantities in eq (18), the errors are

1. The relative uncertainty of the pressure ratio,
  $$\delta \left(\frac{P_{2}}{P_{1}}\right)/\left(\frac{P_{2}}{P_{1}}\right).$$
  The error has two main components, an imprecision depending upon the regulation of the pressure and the variability of the diaphragm, and a fixed uncertainty that can be evaluated from the imprecisions in p, *g* and *h*. The pressure difference between the gas thermometer and the manometer was measured by the diaphragm transducer before and after the bath temperature readings. The estimates of the standard deviation of the mean were calculated from the deviations for the experimental pressures at 100 and 457 °C and also at the fiducial point.
  For 457 °C, the estimate of the standard deviation of the gas thermometer temperature from this source, for a pressure of 10^5^ Pa is ±0.23 mK_GT_. To this should be added a possible random fluctuation of the regulated pressure, estimated in terms of a variance from the manometer setting. This is estimated to amount to ±0.033 Pa at 1 atm (101,325 Pa) and 0.02 Pa at 3.5×10^4^ Pa. These effects combined in quadrature with the variability of the diaphragm are, for one standard deviation, 0.33 mK_GT_ at 10^5^ Pa and 0.52 mK_GT_ at 3.5×10^4^ Pa.
  For 100 °C, the estimate of the standard deviation of the mean of the diaphragm readings affects the gas thermometer temperature by only 0.34 mK_GT_ for 10^5^ Pa, and 0.17 mK_GT_ at 1.8×10^4^ Pa. An estimate of random fluctuation in gas thermometer temperature, from the manometer setting at 10^5^ Pa of 0.033 Pa is equivalent to 0.12 mK_GT_ and at 1.8×10^4^ Pa of 0.02 Pa is equivalent to 0.41 mK_GT_. These errors combined in quadrature are 0.12 mK_GT_ for 10^5^ Pa and 0.44 mK_GT_ for 1.8×10^4^ Pa.
  The total uncertainty of the pressure ratio at the manometer is discussed in [5]. It was stated to be ±1.5 ppm at the 99 percent confidence level, or about 0.5 ppm for 1 standard deviation of the mean. The error can be evaluated from measured quantities, so is of a “B” type.
2. The contribution of the pressure head to the measured pressure in the present temperature range varies between 4.5 ppm and 11.5 ppm and is unlikely to be in error by more than 2 or 3 percent. Therefore, the error of the pressure head correction is relatively insignificant.
3. The errors in the thermal expansion measurements used to calculate the thermal expansion of the bulb. It is believed that the systematic effects are relatively small. Therefore, the error can be evaluated from the imprecision of the measurements, and is of type “B”. The volume expansion is calculated from
  $$V/V_{0}{\left(1+{\bar{\alpha }}_{0}{\;}^{t}\text{Δ}t\right)}^{3}$$ (19)
  where
  $${\bar{\alpha }}_{0}{\;}^{t}=\frac{1}{l_{0}}\frac{l-l_{0}}{t_{68}},$$ (20)
  *l*_0_ is the length at 0 °C, and *t*_68_ is the temperature at which *l* was determined. As stated in section 6, a fifth order power series was fitted by least squares to the data. The estimate of the standard deviation of a predicted point varied from a minimum of 0.8 part in 10^7^ from 0 to 54 °C, 1.9 part in 10^7^ at 457 °C, and was 1 part in 10^7^ at 100 °C. Therefore, the error contributed to the thermodynamic temperature, expressed as the estimate of a standard deviation is 0.38 ppm at 100 °C and 0.62 ppm at 457 °C.
4. The uncertainty in the dead space calculation is primarily due to the uncertainty in the determination of temperatures along the connecting tube. These were measured by thermocouples, the measurement problems of which have been discussed earlier in this paper. The effect of the temperature uncertainty can be evaluated from an approximation for the value of *Vτ*, which is
  $$V_{\tau }=\sum_{k}\frac{V_{k}}{T_{k}}\propto V/\bar{T}$$ (21)
  where $\bar{T}=(t+23)/2+273.15$ is the absolute temperature averaged between the temperatures of the room and the thermostat. We have evaluated the error in the gas thermometer temperatures resulting from a total uncertainty in $\bar{T}$ of *δt*= ±0.4%×(*t*−23) °C. For instance at 457 °C, the average temperature, $\bar{T}$, is assumed uncertain by 1.74 °C for the measurements (corresponding to an uncertainty of about 2.5 °C at *t* itself). A summary of the error calculations, which become insignificant below 141 °C, is given in table 8.

The value of one standard deviation of the mean, *S_m_*, is about ⅓ of the total uncertainty given in the final column.

The remaining sources of error come from uncertainties in the evaluation of thermomolecular pressure and gas imperfection effects. The thermomolecular pressure correction was largest for states 10.01 and 10.02 at 457 °C, where it amounted to 16.4 mK_GT_. The only significant error in the correction derives from the imprecision of the measurements for determining thermomolecular pressure. The estimate of the standard deviation of the mean for the results at each temperature measurement was 1.1 percent at 140.5 °C, 0.68 percent at 260 °C, 1.1 percent at 374 °C and 0.92 percent at 457 °C. The error is of type B, and amounts to about 1 percent of the calculated correction, which in general is not significant.

The effects of gas imperfection were calculated from the virial coefficients for helium, selected as we described earlier. The largest correction for gas imperfection was made for states 9.04 and 9.05 at 400 °C, but it was almost as large for the numerous measurements at 457 °C with *p* = 10^5^ Pa. We postulate that the instrument error in the measurements to evaluate *Z=pV/RT*=1+*Bp/RT* produces a total uncertainty in *Z* of at least ±5 ppm, and is that small only for exceptional work. On the basis of a constant error in the virial coefficient of ±0.11 cm^3^/mol, this in turn makes an error for the combination of *B*_2_ *− B*_1_ of ±0.16 cm^3^/mol, which is perhaps that small only in the range 0–150 °C. At least twice that total uncertainty should be assumed for the measurements at 400 °C (states 9.04 and 9.05), and for the measurements at 457 °C. These uncertainties amount to ±4.4 mK for states 9.04 and 9.05 and ±3.7 mK for the higher pressure measurements at 457 °C.

We can summarize the errors in the thermodynamic temperature as being

$$S_{m}(T)=S_{m}(\text{GT})+S_{m}(\text{Thermp})+S_{m}(\text{Imp})$$ (22)

where *S_m_*(GT) is the estimate of the standard deviation of the mean of the gas thermometer temperature, *S_m_* (Thermp) is the estimate of the standard deviation of the mean of the thermomolecular pressure correction, and *S_m_* (Imp) is the estimate of the standard deviation of the mean of the gas imperfection correction.

The estimate of the standard deviation of the mean of the gas thermometer temperature can in turn be broken up into

$$S_{m}(\text{GT})=S_{m}(\text{PR})+S_{m}(\text{TE})+S_{m}(\text{DS})$$ (23)

where *S_m_* (PR) is the estimate of the standard deviation of the mean of the gas thermometer temperature produced by errors in the pressure ratio.^7^

*S_m_* (TE) is the estimate of the standard deviation of the mean of the gas thermometer temperature produced by errors in the values of the thermal expansion of the bulb.

*S_m_* (DS) is the estimate of the standard deviation of the mean of the gas thermometer temperature produced by errors in the evaluation of the correction for the deadspace.

The total can be found by addition in quadrature of the values given in table 9.

Thus the estimate of the standard deviation of the mean of *T/K−T*_68_*/K*_68_ is the combination of *A, B*, and *C* from tables 7 and 9, as follows:

The observed imprecision should be consistent with the sum of “A” of table 10 and *S*_4_ of table 7 (the observed systematic deviation from work hardening of the *PRT’*s). These latter are, in quadrature, the following:

5.2×10^−4^ at 10^5^ Pa and 457 °C
6.6×10^−4^ at 3.5×10^4^ Pa and 457 °C
1.9×10^−4^ at 10^5^ Pa and 100 °C
4.8×10^−4^ at 1.8×10^4^ Pa and 100 °C.

The corresponding “observed imprecision”, the standard deviation of the mean of the experimental values of *T/K*−*T*_68_*/K*_68_, at each temperature is

6.6×10^−4^ for 10^5^ Pa and 457 °C
1.02×10^−3^ for 10^4^ Pa and 100 °C.
  The large experimental value at 100 °C may indicate sources of random error that were not adequately appraised (as discussed in [8]). The observed imprecisions can also be evaluated more broadly in terms of the deviations of the 72 values of *T/K*−*T*_68_*/K*_68_ from the least-squares fit. The standard deviation of a predicted point for *T/K*−*T*_68_*/K*_68_ is 4.6×10^−4^ at 457 °C and 3.5×10^−4^ at 100 °C.

The estimates of the total uncertainty can be derived by adding the estimates of type A and B in quadrature, and multiplying by 3 as the approximate factor from Student's Table for a 99 percent confidence level. The systematic errors, part *C*, are similarly increased, because they were arbitrarily stated at ⅓ of their estimated values in order to compare them with the values of Types A and B. The summed values for 457 and 100 °C are given in table 11.

## 12. Discussion

All data, including those published previously, have been interpreted in the light of further information and improved understanding of the apparatus. The reevaluation of the volume of the bulb makes an appreciable difference in the dead space calculation for the data published previously (amounting to as much as 2.8 mK at 414.75 K). In contrast, the difference in composition of the top of the bulb from the remainder causes no net change in bulb volume with temperature (compared with an all 88 percent Pt–12 percent Rh composition) in excess of 1 ppm, because the shape of the distortion is compensating in the volume to the first order. The problem is minimized because the total thermal expansion of the two alloys becomes equal for *t*~360 °C. Inasmuch as we had used the values for 88 percent Pt–12 percent Rh in the previous calculations there is no error exceeding 1 ppm from this cause in any of the results, old or new.

All the evidence of system cleanliness—RGA scans, diaphragm, and gas thermometer stability, reproducibility before and after gas thermometer bakeouts—supports the proposition that the results are free of significant effects of sorption (except set G which was not included in the final results). Above 0.01 °C, gas thermometer temperatures are increased by the effects of sorption. It is not generally recognized (although it was mentioned in [9]), that our earliest measurements yielded “thermodynamic temperatures” in excess of 373.15 K at the normal boiling point of water; these values declined as the gas thermometer became cleaner. We believe we could adjust the purity of the gas thermometer to produce a selected value of the steam point over a range of about 0.1 K. In the less clean condition, we expect that there would be less precision and stability, but the gas thermometer would probably be deceptively stable even with enough sorption effect to give “*T*_th_” = 373.15 K.

Operating in the higher temperature range emphasized certain problems, viz., the measurement and treatment of dead space temperatures and of thermomolecular pressures, the evaluation of the effects of gas imperfection, and the realization of international temperatures, all of which can be handled better in the future with higher resulting accuracy. We have installed a new gas thermometer that has a much improved thermocouple installation for measuring the dead space temperatures. Special annealing and testing equipment was built to assure that these couples were uniform and had emf’s in close agreement with accepted values. The effects of thermomolecular pressure and gas imperfection are both encountered in any of our gas thermometer measurements, but they can be evaluated with little loss of accuracy by a modification of the customary measuring techniques:

1. Independent measurement of thermomolecular pressures. The results of some measurements have been reported. Further work is planned where conditions are more exactly defined and more accurate values are obtained at lower average pressures.
2. Use of relatively small fiducial pressures. Our ability to determine the virial coefficients is limited by our ability to determine thermomolecular pressures. Therefore, the highest accuracy results when only one factor is involved. The measurement of thermomolecular pressures and the theoretical justification for the transfer of results to the gas thermometer will be the subject of a separate paper.

It has recently become possible to operate the gas thermometer with no loss of accuracy in a pressure range much lower than was the limit ten years ago. Of special benefit in this respect is the ten-fold improvement in the calibration of gage blocks. No new manometer errors become significant in a low range of pressure based on a fiducial value of 2.54 cm of Hg. The great advantage of this lower pressure range is that the correction for gas imperfection is sufficiently small that the uncertainty is less than 1 ppm at 457 °C for a pressure of 6.78 cm Hg, and the thermomolecular pressure correction, though large, is measurable with such accuracy that its uncertainty is about the same.

## 13. Summary and Conclusions

The deviation of international temperatures from thermodynamic temperatures has been evaluated by a constant volume helium gas thermometer between 0 and 457 °C. Previously reported values were modified for a correction in the dead space effect, and also in light of a reinterpretation of the effects of gas imperfection and thermomolecular pressure. The results are consistent with independent measurements of each of these effects. As examples, the total uncertainty, consisting of the estimate of the random errors at the 99 percent confidence limits and the systematic errors, intended to be conservatively enough stated to warrant about the same confidence, was calculated for each of two pressures at 100 and 457 °C. The estimate of the systematic error in each case was appreciably larger for the higher pressure range, but the results agree with those measured in a lower pressure range. Hence the uncertainties for the lower pressures are regarded as the proper ones, and are given following the value of the deviation:

**Table:** 

*t*	*T/K–T*_68_/*K*_68_

457 °C	−0.0794 ±2.8 × 10^−3^ random error ±3.1 × 10^−3^ systematic error, and
100 °C	−.0252 ± 1.8 × 10^−3^ random error ±.54 × 10^−3^ systematic error

The errors at temperatures between 100 and 457 °C ought to be approximately proportional to the temperature so that one obtains:

**Table:** 

At the tin point, *t*_68_ = 231.9681 °C	

*T/K–T*_68_/*K*_68_= −0.0439	±2.2×10^−3^ random error ±1.5×10^−3^ systematic error

At the zinc point, *t*_68_ = 419.58 °C	

*T/K–T*_68_/*K*_68_= −0.0658	±2.8×10^−3^ random error ±2.8×10^−3^ systematic error

Improved equipment and improved procedures are expected to substantially lower the uncertainties in the next set of measurements, which have been commenced.

## Figures and Tables

**Table 1:** Designations of sets, with added comments

Set	Period	Pressure range	Other comments

A	Nov. 15–Dec. 5, 1973.	Intermediate	Effects of creep.
B	Jan. 15–Feb.1, 1974	Intermediate	
C	Feb. 6–Feb. 19, 1974	Intermediate	
D	Feb. 20–Mar. 5, 1974	High	
	
	Gas thermometer vacuum pumped at 457 °C May 10– May 15, 1974		
E	May 16–June 6, 1974	High	
	
	Resistance thermometers calibrated June 1–June 14, 1974		
F	June 24–July 1, 1974	Low	
G	July 6–Aug. 8, 1974	Intermediate	Suspected contamination.
	
	Gas thermometer vacuum pumped at 457 °C August 9–August 12, 1974		
H	Aug. 13–Aug. 30, 1974.	Intermediate	
	
	Gas thermometer vacuum Dumped at 920 °C August 31–September 11, 1974		
I	Sept. 12–Sept. 13, 1974.	Intermediate	Change of bulb volume.

**Table 2:** Gas thermometer results, sets A, B, C and D

1	2	3	4	5	6	7	8	9	10	11	12	13	14	15	16

State	Ave bath *t* °C	701	703	707	Vault °C	*C*6 *μ*V	*C*7 *μ*V	*C*11 *μ*V	π	*Vτ* mm^3^*/K*	Gage block in	*Z* cm	*T′*_GT_*/K*_GT_	Σ *δt T*_GT_*/K*_GT_ − *T*_68_*/K*_68_	*T/K*−*T*_68_*/K*_68_

2.04	199.57969	8035	7965	7907	19 9179	575	19	26	7.60	0 843234	19.6000184	50.098428	−0.0468	−0.0462	−0.0390
11.01	167.81883	1936	1882	1832	19.9868	607	15	29	8.03	.879305	18.3000156	46.732459	−.0442	−.0438	−.0381
1.12	.05212	5216	5187	5234	19.9569	611	0	41	10.95	1.143320	11.3926111	28.955979	−.00002	−.0002	−.0002
1.13	.00282	271	307	268	19.9797	611	0	41	10.96	1.144712	11.3906113	28.950756	Fiducial	……….	……….
6.03	327.25501	5619	5644	5239	20.0016	547	35	14	6.51	.730605	24.8000249	63.628995	−.0601	−.0587	−.0444
8.04	391.69635	9901	9732	9272	20 0017	559	45	7	6.00	.677523	27.4080254	70.456473	−.0836	−.0821	−.0634
10.02	457.23666	4089	3804	3107	20.0045	587	54	0	5.67	.644306	30.0483289	77.400202	−.1092	−.1075	− 0842
10.03	457.23976	4431	4036	3459	20.0285	575	54	0	5.67	.644577	30.0483289	77.400044	−.1138	−.1121	−.0888
10.04	457.24714	4748	5327	4066	20.0225	560	54	0	5.68	.645384	30 0483289	77.400303	−.1188	−.1170	−.0937
10.05	457.23765	3943	3835	3518	20.0272	560	54	0	5.67	.644916	30.0483289	77 399842	−.1136	−1119	−.886
10.06	457.23818	4196	4074	3185	20.0058	500	54	0	5.68	.646497	30.0483989	77.400268	−.1101	−.1084	−.0851
9.03	424.93184	3321	3360	2870	20.0049	500	48	3	5.88	.667050	28.7480296	73.977531	−.0976	−.0960	−.0751
9.04	424.93024	3153	3209	2709	20.0042	500	48	3	5.87	.665542	28.7480296	73.977283	−.0984	−.0967	−.0758
8.05	391.71112	1223	1222	891	20.0034	480	45	7	6.07	.687159	27.4080254	70.457598	−.0878	−.0863	−.0676
10.07	457.23859	4473	3692	3411	19.9553	667	51	0	5.65	.642461	30.0483289	77.400091	−.1009	−.0992	−.0759
9.05	424.92780	2889	2977	2473	19.9956	639	49	3	5.84	.661441	28.7480296	73.976691	−.0907	−.0891	−.0682
8.06	391.71202	1381	1263	961	19.9858	635	45	7	6.05	.683365	27.4080254	70.457391	−.0803	−.0788	−.0601
7.03	359.55953	6005	6244	5610	19.9158	635	39	10	6.26	.704933	26.1080274	67.050862	− 0692	−.0679	−.0514
6.04	327.27613	7628	7866	7344	19.9305	627	36	14	6.50	.729316	24.8000949	63.629693	−.0654	−.0641	−.0498
6.05	327.27642	7648	7867	7409	19.9298	627	36	14	6.50	.729117	24.8000249	63.629769	−.0649	−.0637	−.0494
5.06	295.24589	4676	4682	4409	19.9887	667	31	17	6.74	.752268	23.5000240	60.235626	−.0589	−.0578	−.0454
4.05	263.30187	295	171	94	19.9734	615	27	20	7.02	.780777	22.2000212	56.850078	−.0583	−.0573	−.0468
3.04	231.41455	1508	1502	1355	19.9860	579	23	23	7.33	.811778	20.9000191	53.470746	−.0558	−.0549	−.0461
1.14	2.40786	787	762	810	19.9939	500	0	40	10.91	1.14086	11.4900096	29.205208	−.00001	−.00001	+.00003
1.15	−.00855	851	874	841	20 0051	524	0	40	10.97	1.14563	11.3900086	28.949104	Fiducial	……….	……….
3.05	231.41455	1570	1449	1345	20.0092	599	22	23	7.35	.814564	20.9000191	53.470813	−.0551	−0542	−.0454
2.05	199.49531	9505	9614	9472	19.9819	587	18	26	7.72	.851899	19.6000184	50.098911	−.0506	−.0500	−.0423
11.02	167.82802	2895	2784	2728	19.9700	587	15	29	8.02	.879258	18.3000156	46.723628	−.0449	−.0446	−.0389
1.16	.08860	8860	8849	8870	19.9863	555	0	40	10.10	1.14442	11.3940123	28.959419	.00017	.00017	.00017
10.08	457.27365	7427	7405	7262	19.9872	647	54	0	5.67	.644047	30.0483289	77.400303	−.1050	−.1033	−.0800
10.09	457.27530	7767	7593	7229	19.9846	647	54	0	5 67	.644099	30.0483289	77.400310	−.1066	−.1049	−.0816
10.10	457.27417	7428	7685	7137	19.9864	639	54	0	5.67	.644346	30.0483289	77.400339	−.1052	−.1034	−.0801
10.11	457.27320	7314	7602	7043	19.9864	639	54	0	5.67	.644343	30.0483289	77.400290	−.1047	−.1029	−.0796
9.06	424.96377	6803	6335	5993	19.9716	627	49	3	5.85	.663317	28.7480296	73.977511	−.0913	−.0896	− 0687
8.07	391.74159	4258	4223	3998	19.9660	587	45	7	6.06	.685320	27.4080254	70.457874	−.0790	−.0775	−.0588
7.04	359.57478	7510	7576	7349	20.0132	579	40	10	6.28	.706876	*26.1080274*	67.049888	−.0686	−.0672	−.0507
6.06	327.29613	9601	9679	9559	19.9712	575	36	14	6.51	.730882	24.8000249	63.629483	−.0635	−.0622	−.0479
1.17	.02188	2221	2182	2160	20.1028	500	0	40	10.98	1.14667	11.3910128	28.951180	Fiducial	………	……….
12.01	400.06997	7221	6941	6827	20.0582	619	45	6	6.04	.683636	36.0000341	92.567811	−.0873	−.0865	−.0617
12.02	400.07089	7205	7250	6812	20.0345	635	45	6	6.02	.681597	36.0000341	92.567881	−.0877	−.0869	−.0621
13.01	323.87915	7933	8026	7785	20.0446	615	36	14	6.55	.734768	32.0000308	82.093154	−.0658	−.0653	−.0470
14.01	248.07564	7583	7615	7496	20.0355	619	27	22	7.17	.796473	28.0000326	71.669921	− 0578	−.0571	−.0444
15.01	172.63305	3289	3369	3256	20.0243	595	17	29	7.97	.874040	24.0000336	61.296616	−.0479	− 0473	−.0403
1.18	.12744	2718	2756	2757	20.0106	575	−1	36	10.95	1.14332	14.7860234	37.580545	Fiducial	……….	……….

Identification of columns: 1 = arbitrary designation of a gas thermometer measurement, 2 = average *t*_68_ determined by 3 *PRT’*s, 3, 4, 5 last 4 digits of *t*_68_ as determined by the thermometer designated by 701, 703, 706, or 707, 6 = vault temperature, 7, 8, 9 = thermocouple readings as discussed in text, 10 = *π*, relative pressure head effect, 11 = value for dead space Σ*_k_V_ki_/T_ki_*, 12 =gage block values used in manometer, 13= Z, augmented pressure expressed in cm of Hg, 14=difference between values found from gas thermometer and *PRT*’s, 15 = difference of values in 14 corrected for a pressure head difference between baths, and the thermomolecular pressure effect, 16 = the difference of values from 15 further corrected for the effects of gas imperfection.

**Table 3:** Gas thermometer results, sets E, F, G, H and I

1	2	3	4	5	6	7	8	9	10	11	12	13	14	15	16

State	Ave Bath *t* °C	701	703	707	Vault °C	*C*6 *μV*	*C*7 *μV*	*C*11 *μV*	π	*Vτ* mm^3^*/K*	Gage blocks in	*Z* cm	*T′*_GT_*/K*_GT_−*T*_68_*/K*_68_	*T*_GT_*/K*_GT_−*T*_68_*/K*_68_	*T/K*−*T*_68_*/K*_68_

9.04	400.07781	7938	7913	7493	19.9122	1049	50	3	5.84	0.656597	36.0000180	92.566491	−0.0863	−0.0855	−0.0607
9.05	400.07842	8163	7785	7577	19.9145	1041	50	14	5.84	.656825	36.0000180	92.566547	−.0882	−.0874	−.0626
8.01	323.88316	8329	8433	8185	19.9505	1007	39	27	6.37	.710510	32.0000120	82.091827	−.0620	−.0614	−.0431
7.01	248.08543	8546	8605	8478	19.9266	989	27	32	7.00	.773604	28.0000080	71.669463	−.0555	−.0551	−.0424
6.01	172.64554	4527	4590	4546	19.8996	921	20	45	7.82	.853059	24.0000090	61.296727	−.0458	−.0455	−.0380
1.01	.21285	1298	1260	1298	19.4946	841	4	79	10.89	1.13583	14.7900210	37.591184	−.00009	−.00009	−.00009
1.02	.02653	2657	2662	2641	19.9315	853	5	82	10.90	1.13647	14.7800240	37.565563	Fiducial	……….	……….
5.01	140.68118	8090	8143	8121	19.9331	883	21	67	8.23	.893283	22.3000130	56.902358	−.0373	−.0371	−.0313
10.01	457.29908	9784	x000	9939	19.9697	1153	60	2	5.49	.620079	10.5510989	27.177196	−.1042	−.0872	−.0790
10.02	457.29983	9816	x333	9801	19.9738	1158	62	−1	5.49	.619983	10.5510989	27.177176	−.1055	−.0885	−.0803
11.01	379.56253	6289	6353	6117	19.9944	1096	49	16	5.97	.670790	9.4508962	24.285564	−.0757	−.0617	−.0555
11.02	379.56304	6331	6412	6170	19.9939	1093	49	20	5.96	.668803	9.4508962	24.285539	−.0769	−.0629	−.0567
1.03	.06066	6070	6050	6078	19.9752	914	3	62	10.88	1.13410	4.0000033	10.166566	Fiducial	………	……….
10.03	457.29595	9467	9880	9437	20.0108	1318	57	−25	5.47	.617593	30.0483217	77.396673	−.0960	−.0889	−.0656
10.04	457.29569	9559	9732	9417	20.0127	1318	58	−19	5.46	.616579	30.0483217	77.396573	−.0913	−.0896	−.0663
10.05	457.29379	9446	9548	9143	20.0123	1321	55	−46	5.47	.617047	30.0483217	77.396510	−.0900	−.0883	−.0660
12.01	424.97992	8277	8039	7662	20.0264	1302	52	−49	5.66	.636436	28.7480235	73.973390	−.0785	−.0769	−.0560
13.01	391.76711	6662	6968	6503	20.0098	1231	46	−26	5.86	.657983	27.4080211	70.454443	−.0723	−.0708	−.0521
14.01	359.61197	1100	1368	1122	19.9801	1202	40	−5	6.08	.679571	26.1080190	67.047804	−.0640	−.0626	−.0460
15.01	327.32650	2646	2677	2627	19.9761	1228	39	−16	6.30	.701470	24.8000160	63.626934	−.0596	−.0584	−.0441
16.01	231.46721	6677	6738	6747	19.9887	1128	24	−13	7.15	.786948	20.9000176	53.469426	−.0519	−.0510	−.0422
16.02	231.46773	6751	6776	6792	19.9878	1129	24	−1	7.15	.786771	20.9000176	53.469488	−.0518	−.0509	−.0421
17.01	136.16997	6928	7004	7058	19.9771	1085	12	12	8.26	.893907	17.0000212	43.372362	−.0358	−.0355	−.0311
1.04	.02701	2679	2715	2709	19.9949	1040	−1	24	10.85	1.12928	11.3900096	28.948912	Fiducial	………	……….
10.06	457.29574	9756	9418	9549	19.9837	1165	51	−11	5.51	.623306	30.0483217	77.397914	−.1017	−.1000	−.0767
10.07	457.29670	9884	9768	9358	19.9867	1176	52	−12	5.51	.622538	30.0483217	77.397748	−.1043	−.1025	−.0792
10.08	457.29657	9844	9777	9350	19.9857	1179	52	−11	5.50	.622227	30.0483217	77.397772	−.1039	−.1022	−.0789
12.02	424.97991	8042	8006	7926	19.9913	1213	46	−27	5.68	.639977	28.7480235	73.974725	−.0879	−.0863	−.0654
13.02	391.76040	5917	6261	5943	19.9937	1190	41	−28	5.88	.660398	27.4080291	70.455153	−.0798	−.0783	−.0596
14.02	359.60084	1	126	124	19.9980	1171	37	−22	6.09	.682020	26.1080270	67.048033	−.0706	−.0693	−.0528
15.02	327.31830	1827	1782	1882	20.0035	1172	30	−20	6.36	.709625	24.8000240	63.627550	−.0645	−.0632	−.0490
18.01	295.29733	9749	9761	9689	19.9961	1154	27	−21	6.61	.734450	23.5000305	60.234739	−.0589	−.0577	−.0453
19.01	263.34529	4570	4505	4513	19.9825	1175	22	−20	6.87	.760698	22.2000277	56.848808	−.0572	−.0562	−.0456
20.01	231.45545	5538	5528	5568	19.9956	1155	20	−6	7.17	.789847	20.9000256	53.469639	−.0540	−.0531	−.0443
21.01	199.62867	2848	2866	2886	20.0017	1156	16	−7	7.50	.821595	19.6000301	50.097231	−.0500	−.0494	−.0417
1.05	.01667	1671	1654	1677	20.0060	1105	−3	3	10.83	1.12683	11.3900096	28.948732	Fiducial	……….	……….
21.02	199.62796	2762	2790	2836	19.9792	1075	23	−9	7.48	.819122	19.6000301	50.098373	−.0503	−.0497	−.0420
1.06	.01724	1734	1727	1711	19.9817	990	−3	37	10.86	1.13133	11.3900096	28.949522	Fiducial	………	………

**Table 4:** Gas thermometer results previously published [10]

1	2	3	4	5	6	7	8	9	10	11	12	13	14	15

State	Ave bath *t* °C	701	703	707	Vault °C	*π*	*Vτ* mm^3^/K	Gage blocks in	*Z* cm	*T′*_GT_*/K*_GT_ − *T*_68_*/K*_68_	*δt*_bulb_ K	*δt*_therm_ K	*δt*_imp_ -K	*T/K*− T_68_*/K*_68_

2.02	100.00097	0093	0065	0133	20.0101	8.63	0.723352	30.0496312	76.569444	−0.0325	+ 0.0019	0.0001	0.0058	−0.0247
1.03	−.00468	464	485	455	20.0128	10.64	.836102	22.0592231	56.053471	Fiducial	…….	…….	…….	…….
2.03	99.99979	9972	9995	x002	20.0381	8.62	.721825	30.0496312	76.568881	−.0330	.0019	.0001	.0058	−.0252
1.04	−.00460	464	477	441	20.0239	10.64	.835937	22.0592231	56.053319	Fiducial	…….	…….	…….	…….
1.05	−.00066	59	86	55	20.1096	10.64	.835661	3.9999996	10.164256	Fiducial	…….	…….	…….	…….
2.04	100.00427	423	397	461	20.0166	8.64	.724165	5.4888939	13.884388	−.0319	.0019	.0041	.0010	−.0249
3.01	120.78497	8478	8464	8548	20.0204	8.30	.701367	5.7488897	14.567342	−.0406	.0023	.0050	.0013	−.0320
4.01	141.59376	9364	9334	9429	20.0146	8.03	.685585	6.0488946	15.431416	−.0473	.0028	.0057	.0016	−.0372
2.05	100.00536	515	511	581	19.9973	8.60	.719052	5.4488939	13.884378	−.0333	.0019	.0041	.0010	−.0263
2.06	10.000484	473	447	531	20.0091	8.59	.718361	5.4489006	13.884336	−.0290	.0019	.0041	.0010	−.0220
3.02	120.79043	935	9011	9084	20.0052	8.30	.701608	5.7488981	14.657444	−.0382	.0023	.0050	.0013	−.0296
4.02	141.59935	9925	9898	9983	20.0029	8.03	.685669	6.0488977	15.431489	−.0455	.0028	.0057	.0016	−.0354
2.07	100.00710	698	681	749	20.0080	8.59	.717602	5.4489006	13.884351	−.0305	.0019	.0041	.0010	−.0235
1.06	.00254	252	243	269	19.9962	10.61	.831801	4.0000047	10.164242	Fiducial	…….	…….	…….	…….
5.01	20.65967	5969	5955	5976	20.0006	10.10	.804239	4.3000022	10.932764	−.0040	.0003	.0010	.0002	−.0025
6.01	41.35574	5582	5556	5583	19.9952	9.64	.779314	4.6002021	11.702679	−.0094	.0007	.0018	.0004	−.0065
7.01	62.06274	6282	6257	6283	19.9905	9.21	.753338	4.9001996	12.472959	.0155	.0010	.0026	.006	−.0113
8.01	82.79370	9344	9333	9434	19.9716	8.90	.738540	5.2000040	13.243896	−.0275	.0015	.00035	.0008	−.0217
2.08	100.01211	1195	1186	1253	19.9975	8.59	.718271	5.4889006	13.884349	−.0344	.0019	.0041	.0010	−.0274
1.07	.00580	574	571	595	19.9929	10.61	.832181	4.0000047	10.164321	Fiducial	…….	…….	…….	…….
5.02	20.66189	6184	6190	6193	19.9816	10.10	.804688	4.3000022	10.932783	−.0045	.0003	.0010	.0002	−.0030
6.02	41.36032	6030	6016	6051	19.9789	9.67	.783029	4.6002021	11.702738	−.0111	.0007	.0018	.0004	−.0082
7.02	62.06529	6521	6519	6546	20.0144	9.24	.756769	4.9001996	12.472967	−.0166	.0010	.0026	.0006	−.0124
7.03	62.06526	6537	6521	6520	20.0145	9.24	.756665	4.9001996	12.472965	−.0167	.0010	.0026	.0006	−.0125
8.03	82.78611	8611	8600	8621	20.0119	8.86	.733769	5.2000040	13.243744	−.0236	.0015	.0035	.0008	−.0178
1.08	.00497	500	479	514	19.9822	10.61	.832388	4.0000047	10.164280	Fiducial	…….	…….	…….	…….

Identification of columns: Columns 1–6 as for table 2, 7 = relative pressure head effect, 8 = value for dead space Σ*_k_*V*_ki_/T_ki_*, 9 = gage block height used in manometer, 10 = *Z*=augmented pressure expressed in cm of Hg, 11 = difference of values found from gas thermometer and PRT’s, 12 = correction required for the revised volume of the bulb, 13 = correction for the thermomolecular pressure effect, 14 = correction for gas imperfection, 15 = difference of thermodynamic and International Practical Kelvin temperatures at the temperature of *t*_68_, given in column 2.

**Table 5:** The second virial coefficient of Helium

*t* (°C)	*B* (cm^3^/mol)

0	12. 00
25	11. 89
50	11. 77
75	11. 67
100	11. 56
125	11. 46
150	11. 36
300	10. 76
400	10. 45
500	10. 14
600	9. 82

**Table 6:** Constants for calculation of T/K−T_68_/K_68_ (eq (15))

*A*_1_= −1.208877838 × 10^5^*K*_68_^2^
*A*_2_= + 1.2135329499 × 10^3^*K*_68_
*A*_3_= −4.3159552
*A*_4_= + 6.4407564676 × 10^−3^ *K*_68_^−1^
*A*_5_=−3.5663884587 × 10^−6^ *K*_68_^−2^

**Table 7:** Errors of realization of t_68_ estimated standard deviation of the mean

Element	t_68_ °C	Type of error		
		*A* mK_68_	*B* mK_68_	*C*^a^ mK_68_

*S*_1_ (Calib.)	457		0.45	
	100		.30	
*S*_2_ (Thermo.)	457	0.20		
	100	.10		
*S*_3_ (Resis.)	457	.02		0.10^b^
	100	.02		.05
*S*_4_ (Hard.)	457			.33
	100			.07
${\sqrt{\Sigma S_{i}}}^{2}$	457	.21	.45	.34
	100	.12	.30	.086

a These values are not to be added with *A* and *B* on the basis that their combination with random errors is not philosophically acceptable. They are stated at 1/3 the value we estimate, for easy comparison with the errors of Type A and B.

b These are values, suggested by experiment, for the ac effects of shunting by dc leads, i.e., a lossy capacitor.

**Table 8:** Estimate of the uncertainty, δt_GT_, in T_GT_ from the assumed uncertainty, δt, in Thermocouple readings

*T* °C_68_	*δt* *K*_68_	$\bar{T}$ *K*_68_	$\delta T/\bar{T}$	$\frac{T_{1}T_{2}}{V_{0}}V\tau \frac{P_{2}}{P_{1}}$ *K*_GT_	δ*T*_GT_ K_GT_

457	1.74	515	0.0034	0.7583	0.0026
425	1.61	499	.0032	.7124	.0023
392	1.48	482	.0031	.6670	.0021
360	1.35	466	.0029	.6248	.0018
327	1.22	450	.0027	.5864	.0016
295	1.09	434	.0025	.5438	.0014
263	.96	418	.0023	.5027	.0012
232	.83	402	.0021	.4621	.0010
200	.71	386	.0018	.4224	.0008
173	.60	373	.0016	.3995	.0006
141	.48	357	.0013	.3522	.0005

**Table 9:** Standard deviations of the mean, and systematic errors in the realization of thermodynamic temperatures

		Class of error		
		A (mK)	B (mK)	C^a^ (mK)

	$457{}^{\circ}C\left\{ \begin{array}{l} 10^{5}\text{Pa}\\ 3.5\times 10^{4}\text{Pa} \end{array}\right.$	0.33		
		.52	0.37	
*S_m_*(PR)	$100{}^{\circ}C\left\{ \begin{array}{l} 10^{5}\text{Pa}\\ 1.8\times 10^{4}\text{Pa} \end{array}\right.$	.12		
		.44	.18	
*S*_m_(TE)	457 °C		.45	
	100 °C		.14	
*S*_m_(DS)	457 °C			0.87
	100 °C			.10
*S_m_*(Thermp)		Negligible		
*S_m_*(Imp)	$457{}^{\circ}C\left\{ \begin{array}{l} 10^{5}\text{Pa}\\ 3.5\times 10^{4}\text{Pa} \end{array}\right.$			1.23
				.43
	$100{}^{\circ}C\left\{ \begin{array}{l} 10^{5}\text{Pa}\\ 1.8\times 10^{4}\text{Pa} \end{array}\right.$			.65
				.12
$\sqrt{\Sigma_{i}S_{i}{\;}^{2}}$	$\left\{ \begin{array}{l} 457{}^{\circ}C\left\{ \begin{array}{l} 10^{5}\text{Pa}\\ 3.5\times 10^{4}\text{Pa} \end{array}\right.\\ 100{}^{\circ}C\left\{ \begin{array}{l} 10^{5}\text{Pa}\\ 1.8\times 10^{4}\text{Pa} \end{array}\right. \end{array}\right.$	.33		1.51
		.52	.58	.97
		.12		.66
		.44	.23	.16

a The errors of Classes A and B are given as one standard deviation. The estimated values of Class C are divided by 3 in order to give a “common basis” of comparison with A and B.

**Table 10:** Estimate of the standard deviation of the mean and of systematic errors in the determination of T/K−T_68_/K_68_

		Class of error		
		A (mK)	B (mK)	C (mK)

457 °C	10^5^ Pa 3.5 × 10^4^ Pa	$\begin{array}{r} 0.39\\ .56 \end{array}\}$	0.73	1.55 1.03
100 °C	10^5^ Pa 1.8 × 10^4^ Pa	$\begin{array}{r} .17\\ .46 \end{array}\}$	.38	.67 .18

**Table 11:** Total uncertainty of T/K−T_68_/K_68_ at 730 and 373.15 K_68_

457 °C	10^5^ Pa	2.7×10^−3^ random error 4.6×10^−3^ systematic error
	3.5×10^4^ Pa	2.8×10^−3^ random error 3.1×10^−3^ systematic error
100 °C	10^5^ Pa 1.8×10^4^ Pa	1.2×10^−3^ random error 2.0×10^−3^ systematic error 1.8×10^−3^ random error .54×10^−3^ systematic error

## Footnotes

1 Figures in brackets indicate the literature references at the end of this paper.

2 The use of commercial product names is for convenience and exactness and does not imply endorsement by the National Bureau of Standards.

3 A new oil diffusion pump has been installed. It is stated by the manufacturer to have a backstreaming rate of “almost zero—less than 5×10^−5^ mg per cm^2^ per min.”

4 The subsequent discussion assumes the gas thermometer was already loaded.

5 Scale comparisons will be given as pure numbers. All temperature scale quantities are therefore divided by their corresponding unit.

6 However, another approach using low pressures corrected by carefully determined thermomolecular pressures is probably better. It is discussed in the final section of this paper.

7 The error of the pressure head is estimated to be insignificant.

## APPENDIX I

100 * FORTRAN IV PROGRAM FOR CALCULATION OF IPTS-68 VALUES
110 * FROM PRT RESISTANCE MEASUREMENTS, NAMED “BRIDGE”
120 DOUBLE PRECISION RT(5), R(5), W, A(10), B(10), T(5), T1, Z
130 DIMENSION N(5)
140 CALL DEFINE (1, 7HTCAL74,)
150 CALL DEFINE (2, 4HPRT,)
160 READ (1, 110)(A(J),E(J),J= 1, 10)
170 110 FORMAT(D15.8,2X,D15.8)
180 120 READ (2, 130) (S1, S2, (N(J),FT(J),R(J),J=1,4))
190 130 FORMAT(2X,F6.2,X,F6.3,(4(/2X,I4,D15.8,D15.8)))
200 WRITE (9, 130)(S1, S2, (N(J), RT(J), R(J), J= 1, 4))
210 WRITE (9, 150) S1, S2
220 150 FORMAT(/14X, ‘STATE’ F6.2,2X,F6.3,/)
230 DO 240 J= 1, 4
240 W=RT(J)/(CR(J)-0.996D-03)
250 I=N(J)-700
260 T(J) = (-A(I)+DSQRT(A(I)**2-4.*(1.-W)*B(I)))/(2.+ B(I))
270 Z = 0.45D-03*T(J)*(T(J)/100.-1.)*(T(J)/419.58-1.)*(T(J)/630.74-1.)
280 T(J)=T(J)+Z
290 WRITE (9,230)N(J),T(J)
300 230 FORMAT(‘THERM.NO.’ I3, 3X, ‘ T(I NT.1968) =’ D17.10)
310 240 (ONTINUE
320 T1 = (T(2) + T(3) + T(4))/3
330 WRITE (9, 270) T1
340 270 FORMAT(‘AVE.T(INT.1968) = ’ D17.10,/,/)
350 GO TO 120
360 END
TCAL74
0.39849575D-02 -0.58762415D-06
0.39850801D-02 -0.58772119D-06
0.39848143D-02 -0.53762185D-06
0.39849917D-02 -0.58769654D-06
0.39849663D-02 -0.58756733D-06
0.39847884D-02 -0.58757724D-06
0.39848898D-02 -0.58760947D-06
0.39850236D-02 -0.58778636D-06
0.39838174D-02 -0.58757363D-06
0.39855387D-02 -0.58755669D-06
PRT
10 9.01 10.036
12 710 0.27587830D 02 0.25557448D 02
14 701 0.63945402D 02 0.25577651D 02
16 703 0.63731295D 02 0.25492645D 02
18 707 0.63956557D 02 0.25582540D 02

## APPENDIX II

100 REM PYE, IN XBASIC, 9.29.75
110 REM USING TWO FILES, DATA AND LSFIT1
115 DBL
120 READ C1, C2, C5, C6
130 DATA 8.74E-6,3E-9,13.0105E-6,.384333E-08
140 DIM Y(18), V(6, 18),G(18),E(18)
150READB0, B1, B2, B3, B4,N(1),N(2),N(3)
160 DATA 135,.545846E-02,.113497E-04,-.152447E-07,.906033 E-11
170DATA1, 4, 12
180FORI= 1TO 13
190PEADG(I)
200NEXTI
210 DATA 62.2,89.6, 116.9, 144.2, 171.6, 198.8,226.3
220 DATA 253.6, 280.9, 308.3, 335.6, 363.0, 390.3
230 FILES DATA,, DISP
240 READ # 1, S0, S1, T9, E0, E1, E2
242 IF END #1 THEM 1090
250 PRINT, 260, S0, S1, T9, E0, E1, E2
260 FMT F7.3, F7.2, F11.5, I6, I5, I5
270 FOR I= 1TO 13
280 READ # 1, E(I)
290 PRINT, 300, E(I),
300 FMT I5
310NEXTI
320 PRINT
330 D2= 0
340 W=0
350 V0=B1*T9 + B2*T9 † 2+B3*T9 † 3+B4*T9 † 4
360 FOR I = 1 TO 13
370 T1 = B0
380 E(I) = 113+(E0-787)*.147+E(I)* 1.00895
390 E(I) =E(I)* 1E-3
400 LET F=B1*T1-E(I)+B2*T1†2+B3*T1†3+E4*T1†4
410 LET G=B1 + 2*B2*T1 + 3*B3*T1†2+4*B4*T1†3
420 T1 = T1-F/G
430 IF ABS(F/G) >2E-4THEN400
440LETD1 = T1+(T1/100)*(-.006+(T1/100-1)*(.04106-7.363E-5*T1))
450LETY (I)= 1/(D1+273, 15)
460NEXTI
470 CALL LSFIT1
480 FOR I=62 TO 144
490 LET X=B(1)+B(2)*I+B(3)*I †2
500 GOSUB 930
510 NEXT I
520 FOR I = 145TO363
530 LET X=B(4)/(I † 2)+B(5)/I + B(6)+B(7)*I + B(8)*I†2
540 GOSUB 930
550 NEXT I
560 FOR I = 364 TO 390
570 LET X=Y(12)+(Y(13)-Y(12))*(I-363)/27
580 GOSUB 930
590 NEXT I
600 IF V0-E(13)>.1 THEN 700
610 FOR I = 391 TO 472
620 LET X=Y(13) + (1/(T9 + 273.15)-Y(13))*(I-390)/82
630 GOSUB 930
640 NEXT I
650 FOR I=473 TO 688
660 LET X= 1/(T9+273.15)
670 GOSUB 930
680 NEXT I
690 GO TO 780
700 FOR I = 391 TO 526
710 LET X=Y(13) + (1/(T9 + 273.15)-Y(13))*(I-390)/136
720 GOSUB 930
730 NEXT I
740 FOR I=527 TO 688
750 LET X= 1/(T9 + 273.15)
760 GOSUB 930770 NEXT I
780 LET D2=D2-55*(C5-C1+(C6-C2)*T3)*T2
790 T(1)=(E0+E1-787)/40+20
800 T(2)=(E0+E2-787)/40+20
810 FOR I = 1TO2
820 T1=T(I)
830 LET D1=T1+(T1/100)*(-0.006+(T1/100-1)*(0.04106-7.363E-5*T1))
840 T(I)=D1+273.15
850 NEXT I
860 W=W+ 13/T(2) + 2*(42-D2)/(T(2) + T(1))+30/(T(D+1/Y(D)
870 LET F=4*980.6/(82.07*1013250)
880 PRINT
890 PRINT“   BOOK   STATE   PI   DISPL”
900 PRINT S0; S1; F*W*.0929, D2*.929
910 PRINT
911 WRITE # 3* D2*.929
920 GO TO 240
930 LET T2=1/X-293.15
940 LET T3 = T2+ 20
950 LET W=W+X* (1 + (C1 + C2*T3)*T2)
960 LET D2=D2+(C5-C1+(C6-C2)*T3)*T2
970 RETURN
980 DATA 10.036,9.05,400.07842, 1041, 50, 14
990 DATA 79,207, 488,849, 1266, 1721,2137,2458,2671
1000 DATA 2846, 2979, 3037, 3073
1090 STOP
1100 END

## APPENDIX III

460LETA9 = 1
470FORA=2TO3
480FORI=N(A-1)TON(A)
490I FN (A) -N (A-1) = 3THEN 520
500LETN=5
510GOTO530
520LETM= 3
530FORK=1 TOM
5401 FM = 3THEN 570
550LETV(K, I)=G(I)†(K-3)
560GOTO 580
570LETV(K, I)=G(I)†(K-1)
580NEXTK
590NEXTI
600LETZ = N(A)
610GOSUB680
620FORL=1TOM
630LETB(A9)=-V(M+ 1,Z+L)
640LETA9=A9+ 1
650NEXTL
660NEXTA
670 RETURN
680FORI=N (A-1)TON(A)
690LETV(M+1, I)=Y(I)
700NEXTI
710FORI = N(A)+1 TON(A)+M
720FORK=1TOM+1
730LETV(K,I) = 0
740NEXTK
750LETV(I -Z, I)=1
760nexti
770fork= 1tom+ 1
780i fk=m+1 then 810
790lett= 1
800goto840
810lett=-1
820goto850
830lett=-t
840i fk= 1 then910
850forl=1tok-1
860lfts(l) = 0
870FORJ =N (A-1) TON(A)
880LETS(L) = S(L) + V(K, J) * V(L, J)
890NEXTJ
900NEXTL
910FORI = N(A-1)TON(A)+M
920I FK= 1THEN 960
930FORL=1TOK-1
940LETV(K,I)=V(K, I) -S(L)*V(L, I)
950NEXTL
960NEXTI
970IFT< 0THEN 1070
980LETD(K)=0
990FORI =N(A-1) TON(A)
1000LETD(K) = D(K) + V(K,I) *V(K, I)
1010NEXTI
1020LETD= SQR(D(K))
1030FORI =N(A-1)TON(A)+M
1040LETV(K,I) = V (K, I)/D
1050NEXTI
10601 FT>0THEN 830
1070NEXTK
1080RETURN
DATA
10 10.018, 10.08, 457.27365, 647, 54, 0
12 86, 223, 511, 915, 1377, 1900, 2392, 2772, 3017, 3243, 3421, 3548, 3665
14 10.018, 10.09, 457.27530, 647, 54, 0
16 83, 219, 507, 916, 1376, 1903, 2401, 2787, 3033, 3257, 3431, 3545, 3654
18 10.018, 10.10, 457.27417, 639, 54, 0
20 90, 230, 519, 924, 1376, 1890, 2373, 2753, 2998, 3228, 3410, 3531, 3649
22 10.018, 10.11, 457.27320, 639, 54, 0
24 88, 227, 512, 919, 1371, 1894, 2387, 2772, 3019, 3244, 3421, 3535, 3646
26 10.018, 9.06, 424.96377, 627, 49, 3
28 78, 200, 455, 829, 1227, 1698, 2145, 2498, 2733, 2944, 3115, 3221, 3314
30 10.018, 8.07, 391.74159, 587, 45, 7
32 71, 180, 405, 728, 1078, 1491, 1889, 2209, 2433, 2635, 2805, 2897, 2990
34 10.018, 7.04, 359.57478, 579, 40, 10
36 63, 160, 359, 640, 948, 1312, 1662, 1950, 2161, 2350, 2513, 2607, 2684

## APPENDIX IV

100 REM DEDSPS, IN XBASIC,9.29.75
110 REM WITH THREE FILES: “DATA, ” GIVING S0, S1, T9, E0, E1,E2 AND
120 REM A1 THROUGH B1; “DATA2, ” LISTING VALUES OF “A” FOR I.D.
130 REM OF TUBE, AND LSFIT1 FOR LEAST SQUARES.
140 DBL
150 READ C1, C2, C3, C4
160 DATA 8.7252E-6, 2.5279E-9, 3.00786E-12, 2.67373E-15
170 DIM Y(18),V(6, 18),G(18),H(18),E(18),P(390)
180READB0, B1, B2, B3, B4/N(1), N(2)/N(3)
190 DATA 135,.545846E-02,.113497E-04,-.152447E-07,.906033E-11
200 DATA1, 4, 12
210 FOR I = 1TO 13
220 READ H(I)
230NEXTI
240 DATA 62.2,89.6,116.9,144.2,171.6,198.8,226.3
250 DATA 253.6,280.9, 308.3, 335.6, 363,390.3
260 FILES DATA, DATA2, DISP
270 FOR I = 1TO 390
280 READ #2,A
290 P(I) = 0.90123+2.748E-4*A
300NEXTI
310 READ # 1,S0,S1,T9,E0,E1,E2
320 IF END #1 THEN 1210
330 READ # 3,Z2
340 PRINT Z2
350 PRINT, 360,S0,S1,T9,E0,E1,E2
360 FMT F7.3,F7.2,F11.5,I6,I5,I5
370FORI=1 TO 13
380 G(I)=H(I)-Z2
390NEXTI
400 H2= 0
410 IF Z2<.5 THEN 450
420 H2=H2+1
430 Z2=Z2-1
440 GOTO410
450 PRINT
460 FOR I = 1 TO 13
470 READ # 1, E(I)
480 PRINT,490,E(I),
490 FMT I5
500NEXTI
510 PRINT
520 V0=B1* T9 + B2* T9 † 2+B3*T9 † 3+B4*T9† 4
530FORI=1 TO 13
540 E(I) = (113+ (E0-787)*.147+E(I)* 1.00895)/1000
550 H1 = E(I)
560 GO SUB 1140
570 Y(I) = 1/(D1 + 273.15)
580NEXTI
590 CALL LSFIT1
600 D1=(E0-787)/40+20
610 D3=E2/40
620 D4 = E1/40
630 T6 = D1 + D3
640 D2=T6-23
650 X7=(1+ (C1 + C2*T6+C3*T6† 2+C4*T6† 3)* D2) †3
660 V5=5.367*X7/(T6+273.15)
670 FOR I= 1TO 7
680 GO SUB 1100
690NEXTI
700 FOR I=8TO42-H2
710 T6=D1+D4+(D3-D4)*(42-H2-I)/(42-H2-8)
720 GO SUB 1100
730NEXTI
740 FOR I=43-H2TO62-H2
750 T6= 1/Y(1) + (D1 + D4+273.15-1/Y(1))*(62-H2-I)/20-273.15
760 GO SUE 1100
770NEXTI
780PRINT
790 FOR I = 63-H2TO 144-H2
800 T6= 1/(B(1)+B(2)*I+B(3)*(I†2))-273.15
810 GO SUB 1100
S20NEXTI
830 FOR I=145-H2TO363-H2
840 T6= 1/(B(4)/(I † 2) +B(5)/I + B(6) + B(7) * I + B(8) *(I † 2))-273.15
850 GO SUB 1100
860NEXTI
870 FOR I = 364-H2TO390-H2
880 T6=L/((Y(13)-Y(12))*(I -(363-H2))/27+Y(12))-273.15
890 GO SUE 1100
900NEXTI
910 I = 390
920 IF V0-E(13) >.1 THEN 1020
930 FOR J= 391-H2 TO 472
940 T6=1/(Y(13) + (1/(T9 + 273.15)-Y(13))*(J-(390-H2))/(82+H2))-273.15
950 GOSUB 1100
960NEXTJ
970 FOR J=473 TO 526
980 T6=T9
990 GOSUB 1100
1000NEXTJ
1010 GO TO 1060
1020 FOR J = 391-H2 TO 526
1030 T6=1/(Y(13) + (1/(T9 + 273.15)-Y(13))*(J-(390-H2))/(136+H2))-273.15
1040 GO SUE 1100
1050NEXTJ
1060 PPIMT“   BOOK   STATE  SUM V*TAU”
1070 PPINT S0;S1;V5
1080 PPINT
1090 GO TO 310
1100 D2=T6-23
1110 X7=(1+(C1+C2*T6+C3*T6† 2+C4*T6† 3)*D2) †3
1120 V5=V5+.735395*P(I)†2*.929*X7/(T6+273.15)
1130RETURN
1140 T1 = B0
1150 F=B1*T1-H1 + B2*T1 † 2+B3*T1† 3 + B4*T1† 4
1160 LET G=B1 + 2*B2*T1 + 3*B3*T1†2+4*B4*T1†3
1170 T1=T1-F/G
1180 IF ABS(F/G)>2E-4 THEM 1150
1190 D1 = T1 + (T1/100)* (-.006+(T1/100-1)*(0.04106-7.363E-5*T1))
1200RETURN
1210 STOP
1220 END

## APPENDIX V

100 * PROGRM “SUMMA”, REVISED APRIL 21, 1975
110 * FORTRAN IV DOUBLE PRECISION, BULE IIIB, DIAPHRAGM IA
120 DOUBLE PRECISION C, C1,C2,C3,C4, V, T, E1, E2, E3, E4
130 DOUELE PRECISION H, H1, T9, PI, V9, D 1, C5, C6
140 DOUBLE PRECISION T1, T2, T3, T4, P, Y, Z, W
150 DATA C,V/0.17255D-03, 0.4325D 06/
160 DATA C1,C2/0.8750894565D-05, 0.2278241267D-08/
170 DATA C3,C4/-0.2476697519D- 11, 0.3461903664D-14/
175 DATA C5/-0.1911694941D-17/
180 (ALL DEFINE (1,6HDATA3,)
190 60 READ(1,65) S0,S1,E1,E2,E3,E4
200 65 FORMAT(4X,F6.3,X,F6.2,X,D9.2,X,D9.2,X,D9.2,X,D9.2)
210 WRITE (9,65) S0,S1,E1,E2,E3,E4
220 READ (1,75) T,H,H 1
230 75 FORMAT (4X,D13.6,X,D13.6,X,D10.3)
240 WRITE (9, 75) T,H,H 1
250 READ (1,85) T9,PI,V9,D1
260 85 FORMAT (4X, D15.8,X, D13.6,X, D13.6, X, D9.2)
270 WRITE (9,85) T9, PI, V9, D1
280 T1 = T+E1/0.4858D 03
290 T2=T+E2/0.1012D 04
300  T3=T+E3/0.4048D 03
310 T4=T+E4,0.4858D 03
320 H=(H + H1* lD-06)*0.254D 01
330 P=-C*H*(T2-0.2D 02)+0.227D-07*H*H-0.1D-03*H
340 H=H+P+0.6085D-03*((T3+T4),0.2D 01-T1) + 0.1108D-04* (T 1-0.2D 02)
350 H=H+ 0.1289D-03+0.1324503D-05*D1
355 C6= C1 + C2*T9 + C3*T9**2+C4*T9**3+C5*T9**4
360 Y=((0,1D 01+C6*T9)**3)-0.1D 01
370 W= PI+Y+ PI*Y+(T9 + 0.27315D 03+0.133053D-02*H)*V9/V
380 Z=H+H*W
390 WRITE (9, 240) S0,S1,Z
400 240 FORMAT (‘ BOOK ’ 4X, ‘ STATE’ 18X, ‘Z’,F6.3,X,F6.2,4X,D17* 10)
410 GO TO 60
420 END
DATA3
10 10.03615.01 -0.47D 02 -0.20D 01 -0.15D 02 -0.24D 02
12 0.199761D 020.248000D 020.160D 02
14 0.32732650D 030.629742D-050.701470D 000.18D 02
16 10.03616.01 -0.50D 02 -0.40D 01 -0.16D 02 -0.25D 02
18 0.199887D 020.209000D 020.176D 02
20 0.23146721D 030.714875D-050.736947D 00 -0.50D 01
22 10.03616.02 -0.50D 02 -0.60D 01 -0.15D 02 -0.25D 02
24 0.199878D 020.209000D 020.176D 02
26 0.23146773D 030.714704D-050.786771D 000.29D 02
28 10.03617.01 -0.51D 02 -0.20D 01 -0.16D 02 -0.27D 02
30 0.199772D 020.170000D 020.212D 02
32 0.13616997D 030.825908D-050.893907D 00 -0.20D 01
34 10.0361.04 -0.53D 020.00D 00 -0.16D 02 -0.27D 02
36 0.199949D 020.113900D 020.960D 01
38 0.27010000D-010.108452D-040.112928D 01 -0.10D 01

## APPENDIX VI

5 DIM Y(90), V(6, 90)
6 DIM B(90), W(90)
7 FILES L5DAT
10 PPINT “INPUT: NO.OF CONSTANTS”
20 INPUT M
110 I = 1
111 GO TO 115
112 I=I+1
115 READ # l,B(I),Y(I)
116 IF END #1 THEN 260
117 B(I)=B(I) + 273.15
120 W(I)= 1
130 V(1, I)=1/B(I)† 2
140 V(2, I) = 1/B(I)
150 V(3,I) = 1
160 V(4, I) =B(I)
170 V(5, I) = B(I) † 2
180 V(6, I) = B(I) † 3
250 GO TO 112
260 N=I- 1
261 PRINT
262 PRINT “N =” N
263 PPINT
270 GOSUE 313
280STOP
313LETN 1 = 0
315FORI=1TON
316LETV(M+1,1)=Y(I)
319LETM 1=N 1+ 1
321NEXTI
333FORI =N+1TON + M
344FORK=1TOM+ 1
355LETV(K,I)=0
366NEXTK
377LETV(I-N,I)=1
399NEXTI
411FORK=1TOM+ 1
4121 FK=M+1THEN417
415LETT= 1
416GOTO422
417LETT=-1
413GOTO433
420LETT=-T
422I FK= 1THEN511
433FORL=1TOK- 1
444LETS(L) = 0
455FORJ=1TON
466 S(L)=S(L)+V(K,J)*V(L,J)*W(J)
477NEXTJ
488NEXTL
511FORI=1TON+M
533IFK=1THEN577
544FORL= 1TOK- 1
555LETV(K,I)=V(K,I)-S(L)*V(L,I)
566NEXTL
577NEXTI
600IFT< 0THEN699
611LETD(K) = 0
622FORI=1 TON
633 D(K) = D(K)+V(K, I)*V(K, I)*W(I)
644NEXTI
655LETD=SQR(D(K))
666FORI=1TON+M
677LETV(K, I)=V(K, I)/D
688NEXTI
693IFT> 0THEN420
699NEXTK
922LETS= 0
933FORI=1TON
944 S=S+V(M+ 1, I) * V(M+ 1, I)*W(I)
955NEXTI
966LETS=SQR(S/(N1-M))
1111PRINT“TERM”, “COEFFICIENT”, “STD.DEV.”
1122PRINT
1133FORL= 1 TOM
1144 LET C(L) =0
1155 FORK=LTOM
1166LETC(L)=C(L)+V(K, N+L)*V(K,N+L)
1177NEXTK
1188LETC(L)=S*SQR(C(L))
1199PRINTL,-V(M+1,N+L>, C(L)
1211NEXTL
1222PRINT
1233PRINT
1244PRINT“STANDARD DEVIATION=”S 1255PRINT
1266PRINT
1277 PRINT“B(I) ”, “Y (I) ”, “Y”, “DEV.”
1288PRINT
1299FORI =1 TON
1311 PRINT B(I);Y(I) ;Y(I)-V(M+ 1, I) ; V(M+ 1, I)
1313 X=X+V(M+ 1, I)
1322NEXTI
1323 PRINT
1324 PRINT “SUM OF RESIDUALS =” X
1333 RETURN
6000 END
1400 457.29, .0790, 457.29, .0803., 457.29, .0800, 457.29, .0801
1410 457.29, .0767, 457.29, .0792, 457.29, .0789, 457.29, .0759
1420 457.29, .0816, 457.29, .0796
1430 424.98, .0654, 424.98, .0687, 424.98, .0632
1440 400.08, .0607, 400.08, .0626, 400.08, .0617, 400.08, .0621
1450 391.76, .0596, 391.76, .0588, 391.76, .0601
1460 379.56, .0555, 379.56, .0567
1470 359.56, .0514, 359.56, .0507, 359.56, .0528
1480 327.32, .0490, 327.32, .0493, 327.32, .0494, 327.32, .0479
1490 323.88, .0431, 323-88, .0470
1500 295.30, .0453, 295.30, .0454
1510 263.35, .0457, 263.35, .0468
1520 248.09, .0424, 248.09, .0447
1530 231.45, .0443, 231.45, .0461, 231.45, .0454
1540 199.63, .0417, 199.63, .0420, 199.63, .0423
1550 172.65, .0380, 172.65, .0403
1560 167.83, .0389
1570 141.59, .0372, 141.59, .0354
1580 120.78, .0320, 120.73, .0296
1590 100, .0247, 100, .0252, 100, .0249, 100, .0263, 100, .0220, 100, .0235
1600 100, .0274
1610 82.79, .0217, 82.79, .0220, 82.79, .0178
1620 62.06, .0113, 62.06, .0124, 62.06, .0125
1630 41.36, .0065, 41.36, .0082
1640 20.66, .0025, 20.66, .0030
1650 2.4079, -.00003
1660 .0886, -.00017
1670 .2129, .00009
1680 .0521, .00002
1690 0.0100, 0

## APPENDIX VII

**Table:**

TEPM	COEFFICIENT	STD.DEV.
1	120887.7838	6691.4096838
2	−1213.5329499	62.65908179
3	4.3159552	.21163095
4	−.64407564676E-02	.30556699962E-03
5	.35663884587E-05	.15950626366E-06

STANDAFD DEVIATION =.15230847863E-02

**Table:**

B(I)	Y (I)	Y	DEV.
730.44	.79000000000E-01	.79392362821E-01	−.39236282133E-03
730.44	.80300000000E-01	.79392362821E-01	.90763717880E-03
730.44	.80000000000E-01	.79392362821E-01	.60763717865E-03
730.44	.80100000000E-01	.79392362821E-01	.70763717862E-03
730.44	.76700000000E-01	.79392362821E-01	−.26923628212E-02
730.44	.79200000000E-01	.79392362821E-01	−.19236282138E-03
730.44	.78900000000E-01	.79392362821E-01	−.49236282130E-03
730.44	.75900000000E-01	.79392362821E-01	−.34923628212E-02
730.44	.81600000000E-01	.79392362821E-01	.22076371787E-02
730.44	.79600000000E-01	.79392362821E-01	.20763717875E-03
698.13	.65400000000E-01	.67446957072E-01	−.20469570721E-02
698.13	.68700000000E-01	.67446957072E-01	.12530429280E-02
698.13	.68200000000E-01	.67446957072E-01	.75304292793E-03
673.23	.60700000000E-01	.60436414255E-01	.26358574537E-03
673.23	.62600000000E-01	.60436414255E-01	.21635857454E-02
673.23	.61700000000E-01	.60436414255E-01	.12635357453E-02
673.23	.62100000000E-01	.60436414255E-01	.16635857454E-02
664.91	.59600000000E-01	.58479128545E-01	.11208714551E-02
664.91	.58800000000E-01	.58479128545E-01	.32037145507E-03
664.91	.60100000000E-01	.58479128545E-01	.16208714551E-02
652.71	.55500000000E-01	.55930437313E-01	−.43043731304E-03
652.71	.56700000000E-01	.55930437313E-01	.76956268638E-03
632.71	.51400000000E-01	.52511784289E-01	−.11117842889E-02
632.71	.50700000000E-01	.52511784289E-01	−.18117842888E-02
632.71	.52800000000E-01	.52511784289E-01	.28821571121E-03
600.47	.49000000000E-01	.48688352293E-01	.31164770716E-03
600.47	.49800000000E-01	.48688352293E-01	.11116477072E-02
600.47	.49400000000E-01	.48688352293E-01	.71164770717E-03
600.47	.47900000000E-01	.48688352293E-01	−.78835229279E-03
597.03	.43100000000E-01	.48383358275E-01	−.52833582746E-02
597.03	.47000000000E-01	.48383358275E-01	−.13833582745E-02
568.45	.45300000000E-01	.46431954863E-01	−.11319548632E-02
568.45	.45400000000E-01	.46431954863E-01	−.10319548632E-02
536.5	.45700000000E-01	.45060865144E-01	.63913485577E-03
536.5	.46800000000E-01	.45060865144E-01	.17391348557E-02
521.24	.42400000000E-01	.44511634947E-01	−.21116349468E-02
521.24	.44700000000E-01	.44511634947E-01	.18836505315E-03
504.6	.44300000000E-01	.43862096791E-01	.43790320916E-03
504.6	.46100000000E-01	.43862096791E-01	.22379032092E-02
504.6	.45400000000E-01	.43862096791E-01	.15379032091E-02
472.78	.41700000000E-01	.42088479685E-01	−.38847968548E-03
472.78	.42000000000E-01	.42088479685E-01	−.88479685445E-04
472.78	.42300000000E-01	.42088479685E-01	.21152031448E-03
445.8	.38000000000E-01	.39573254893E-01	−.15732548930E-02
445.8	.40300000000E-01	.39573254893E-01	.72674510700E-03
440.98	.38900000000E-01	.38990228025E-01	−.90228024703E-04
414.74	.37200000000E-01	.34956871926E-01	.22431280741E-02
414.74	.35400000000E-01	.34956871926E-01	.44312807410E-03
393.93	.32000000000E-01	.30615283123E-01	.13847168776E-02
393.93	.29600000000E-01	.30615283123E-01	−.10152831225E-02
373.15	.24700000000E-01	.25233918747E-01	−.53391874735E-03
373.15	.25200000000E-01	.25233918747E-01	−.33918747361E-04
373.15	.24900000000E-01	.25233918747E-01	−.33391874734E-03
373.15	.26300000000E-01	.25233918747E-01	.10660812526E-02
373.15	.22000000000E-01	.25233918747E-01	−.32339187474E-02
373.15	.23500000000E-01	.25233918747E-01	−.17339187473E-02
373.15	.27400000000E-01	.25233918747E-01	.21660812527E-02
355.94	.21700000000E-01	.20071511472E-01	.16284885281E-02
355.94	.22000000000E-01	.20071511472E-01	.19284885280E-02
355.94	.17800000000E-01	.20071511472E-01	−.22715114719E-02
335.21	.11300000000E-01	.13314065430E-01	−.20140654299E-02
335.21	.12400000000E-01	.13314065430E-01	−.91406542990E-03
335.21	.12500000000E-01	.13314065430E-01	−.81406542992E-03
314.51	.65000000000E-02	.66790537379E-02	−.17905373787E-03
314.51	.82000000000E-02	.66790537378E-02	.15209462622E-02
293.81	.25000000000E-02	.15210989612E-02	.97890103884E-03
293.81	.30000000000E-02	.15210989612E-02	.14789010388E-02
275.5579	−.30000000000E-04	.94616373689E-04	−.12461637369E-03
273.2386	−.17000000000E-03	.25398657250E-03	−.42398657250E-03
273.3629	.90000000000E-04	.24301654224E-03	−.15301654224E-03
273.2021	.20000000000E-04	.25726150310E-03	−.23726150310E-03
273.16	0	.26106913775E-03	−.26106913775E-03

SUM OF RESIDUALS =−.47748471843E-11
The least squares solution presented does not give a calculated value of zero for T_68_/K_68_ − T/K at T_68_ = 273.16 K_68_, but amounts to 0.000261. The equation has a minimum at 278.57 K_68_, where the value is less than 0.000023. However, the variation at 273.16 K_68_ is within experimental error, and can be ignored for any scale adjustments.
